# A workflow for single-particle structure determination via iterative phasing of rotational invariants in fluctuation X-ray scattering

**DOI:** 10.1107/S1600576724000992

**Published:** 2024-03-15

**Authors:** Tim B. Berberich, Serguei L. Molodtsov, Ruslan P. Kurta

**Affiliations:** a European XFEL, Holzkoppel 4, 22869 Schenefeld, Germany; b I. Institute of Theoretical Physics, University of Hamburg, Notkestraße 9-11, 22607 Hamburg, Germany; c Institute of Experimental Physics, TU Bergakademie Freiberg, Leipziger Straße 23, 09599 Freiberg, Germany; d Center for Efficient High Temperature Processes and Materials Conversion (ZeHS), TU Bergakademie Freiberg, Winklerstrasse 5, 09599 Freiberg, Germany; DESY, Hamburg, Germany

**Keywords:** fluctuation X-ray scattering, single-particle imaging, iterative phasing, X-ray free-electron lasers

## Abstract

Fluctuation X-ray scattering (FXS) offers a complementary approach for nano- and bioparticle imaging with an X-ray free-electron laser (XFEL), by extracting structural information from correlations in scattered XFEL pulses. Here a workflow is presented for single-particle structure determination using FXS. The workflow includes procedures for extracting the rotational invariants from FXS patterns, performing structure reconstructions via iterative phasing of the invariants, and aligning and averaging multiple reconstructions. The reconstruction pipeline is implemented in the open-source software *xFrame* and its functionality is demonstrated on several simulated structures.

## Abstract 2

A single-particle structure determination pipeline is implemented in the open-source software *xFrame*, which includes methods for determining rotational invariants from X-ray scattering patterns and performing structure reconstructions by iterative phasing of rotational invariants.

## 1. Introduction

Advances in X-ray sources and instrumentation over recent decades (Jaeschke *et al.*, 2016 ▸) have been accompanied by an extensive development of techniques and methods for X-ray diffraction and imaging (Chapman *et al.*, 2006 ▸; Rodenburg, 2008 ▸; Nugent, 2010 ▸; Nakasako *et al.*, 2020 ▸). The emergence of high-power X-ray free-electron lasers (XFELs) (Ueda, 2018 ▸) opened new horizons for crystallographic studies of biological materials (Chapman *et al.*, 2011 ▸; Boutet *et al.*, 2012 ▸; Wiedorn *et al.*, 2018 ▸), most importantly in the time domain (Pandey *et al.*, 2020 ▸; Orville, 2020 ▸). At the same time, intense and ultrashort X-ray pulses produced by an XFEL made it possible to carry out ‘diffraction before destruction’ experiments on individual bioparticles (Bogan *et al.*, 2008 ▸; Mancuso *et al.*, 2010 ▸; Seibert *et al.*, 2011 ▸; Hantke *et al.*, 2014 ▸; Kimura *et al.*, 2014 ▸; Ekeberg *et al.*, 2015 ▸; Rose *et al.*, 2018 ▸), as it was previously envisioned (Solem & Baldwin, 1982 ▸; Neutze *et al.*, 2000 ▸; Gaffney & Chapman, 2007 ▸). Such experiments open up the possibility of imaging particles for which it is difficult or impossible to obtain crystals. At the same time, while serial crystallography with an XFEL allows for high-resolution structure determination of proteins and macromolecules, single-particle imaging (SPI) is a developing way to provide biologically significant structural information (Aquila *et al.*, 2015 ▸; Chapman, 2019 ▸; Bielecki *et al.*, 2020 ▸).

Several approaches have been proposed so far for structure determination of bioparticles from scattering measurements with an XFEL. The most common approach is based on iterative phasing of the measured single-particle intensity patterns (Fienup, 1982 ▸; Marchesini, 2007 ▸), which enables *ab initio* high-throughput imaging of 2D structure projections (Seibert *et al.*, 2011 ▸; Hantke *et al.*, 2014 ▸; Kimura *et al.*, 2014 ▸). Since, generally, a complete 3D structure of a particle is of interest, it is necessary to assemble the 3D scattered intensity distribution from 2D diffraction patterns, measured from reproducible particles in unknown orientations (Ekeberg *et al.*, 2015 ▸; Rose *et al.*, 2018 ▸; Nakano *et al.*, 2018 ▸; Assalauova *et al.*, 2020 ▸). The latter is known as the orientation determination problem and is often solved using Bayesian methods (Loh & Elser, 2009 ▸; Flamant *et al.*, 2016 ▸) or other approaches (Bortel & Tegze, 2011 ▸; Yefanov & Vartanyants, 2013 ▸; Fung *et al.*, 2009 ▸; Giannakis *et al.*, 2012 ▸; Kassemeyer *et al.*, 2013 ▸; Nakano *et al.*, 2017 ▸). Such a two-step SPI approach, involving orientation determination and iterative phasing, can only be applied to the diffraction snapshots measured from individual particles. This might be challenging to accomplish in practice for arbitrary small bioparticles such as proteins, for which the individual snapshots are weak and noisy (Ekeberg *et al.*, 2022 ▸).

One of the possible ways to study weakly scattering noncrystalline particles is to perform X-ray measurements on a multiparticle system, as realized in biological small-angle X-ray scattering (SAXS) (Chaudhuri *et al.*, 2017 ▸; Vela & Svergun, 2020 ▸). Such solution scattering measurements are, however, associated with loss of information caused by the rotational averaging of intensities from individual particles in the ensemble, and typically result in a low-resolution fit of the particle structure. At the same time, taking solution scattering snapshots by XFEL pulses which are shorter than the characteristic rotational diffusion time of the particles allows one to measure structural information that is usually inaccessible in traditional SAXS at synchrotron sources. This additional information is hidden in the scattered intensity fluctuations defined by an instantaneous configuration of the ensemble of particles, and can be extracted by means of angular cross-correlation functions (CCFs). The fluctuation X-ray scattering (FXS) approach thus seeks to determine the structure of a single particle, by using statistically averaged CCFs measured from a dilute multiparticle system (Kam, 1977 ▸, 1980 ▸; Kam *et al.*, 1981 ▸). Therefore, FXS can potentially bridge the gap between conventional imaging and crystallographic methods.

FXS is a natural extension of SAXS because it also relies on rotationally invariant descriptions of the 3D single-particle intensity distribution (Kurta *et al.*, 2017 ▸). Similarly to SAXS, forward modeling approaches are also applicable to FXS data, where the reciprocal-space constraints are expressed by CCFs (Liu *et al.*, 2013 ▸; Malmerberg *et al.*, 2015 ▸; Kurta *et al.*, 2017 ▸). In fact, the information content of FXS measurements is substantially higher as compared with SAXS. For instance, in the case of 2D structure determination, it has been shown that the information accessible via FXS is equivalent to complete knowledge of the 2D single-particle intensity pattern (Kurta *et al.*, 2013 ▸; Pedrini *et al.*, 2013 ▸). Moreover, despite the limited information content of two-point CCFs (Elser, 2011 ▸), they are sufficient to produce successful *ab initio* 3D structure reconstructions (Donatelli *et al.*, 2015 ▸; Kurta *et al.*, 2017 ▸; Pande *et al.*, 2018 ▸).

Although the idea of biological FXS was formulated almost half a century ago (Kam, 1977 ▸), it was first put into practice only recently with the advent of XFELs (Kurta *et al.*, 2017 ▸; Pande *et al.*, 2018 ▸). Progress in the development of X-ray instrumentation and sample delivery systems has led to a recent surge in FXS-related activity (Wochner *et al.*, 2009 ▸; Altarelli *et al.*, 2010 ▸; Saldin *et al.*, 2010 ▸, 2011 ▸; Kirian *et al.*, 2011 ▸; Kurta *et al.*, 2012 ▸; Starodub *et al.*, 2012 ▸; Mendez *et al.*, 2016 ▸; Martin, 2017 ▸). At the same time, practical applications of FXS are still quite rare compared with the more traditional SPI or SAXS (Kurta *et al.*, 2016 ▸). The availability of relevant practical algorithms and open-source software that implement the quite involved and often obscure mathematical apparatus of FXS may help to advance in this direction. Here we present a workflow for single-particle structure determination via iterative phasing based on rotational invariants which are accessible in FXS. The workflow is implemented in the open-source software suite *xFrame*, which includes methods for computing the CCF, extracting rotational invariants and performing structure reconstructions, as well as subsequent alignment and averaging of multiple reconstructions.

## 2. Theoretical background

### 2.1. Fluctuation X-ray scattering

**Figure 1:**
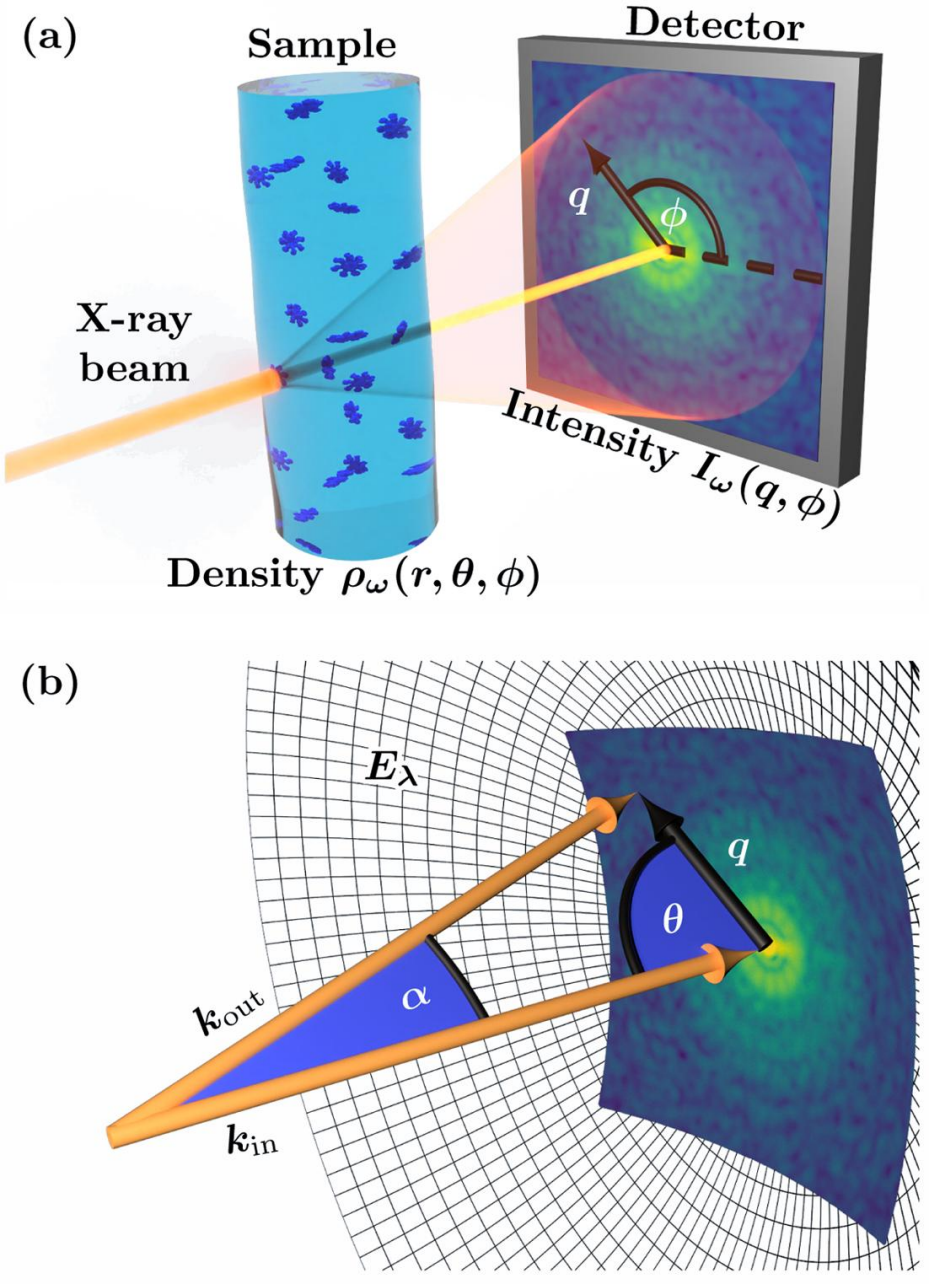
Scattering geometry of an FXS experiment. (*a*) The incident X-ray beam is diffracted from a sample solution (or aerosol) and recorded in the far field on a 2D detector. Here 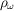 corresponds to the illuminated portion of the dilute sample and 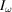 denotes the scattered intensity. (*b*) Measured diffraction pattern mapped on the Ewald sphere 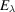 , where 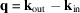 is the scattering vector, 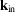 is the wavevector of the incident X-ray pulse, 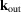 is the wavevector of the scattered pulse and 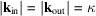 .

We first define the real-space single-particle electron density by ρ(**r**) and the corresponding scattered X-ray intensity distribution in reciprocal space by *I*(**q**), where **r** and **q** are the real- and reciprocal-space vectors, respectively. Within the kinematic X-ray scattering approximation the density ρ(**r**) is related to the scattered intensity *I*(**q**) in the far field via the absolute square of its Fourier transform 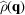 , 

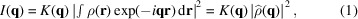

where *K*(**q**) is a **q**-dependent term which encompasses relevant experimental factors, *e.g.* polarization of X-rays, incident intensity fluctuations *etc.* [see, for instance, a review of possible intensity corrections in SAXS experiments (Pawn, 2013 ▸)]. Hereafter, we assume that each experimental image can be properly corrected for *K*(**q**), so that the resulting scaled *I*(**q**) is defined only by the electron density of the sample. We also assume that any background scattering present in realistic measurements (*e.g.* solvent scattering or parasitic scattering from beamline components), which is neglected in equation (1), can also be properly corrected. We can then consider realizations of an ensemble of *N*_p_ ≥ 1 reproducible particles, randomly positioned and oriented in space. Similarly to conventional SAXS from dilute solutions of biological particles (Vela & Svergun, 2020 ▸), we assume scattering conditions such that interference scattering between different particles can be neglected. Furthermore, let us denote the electron density of a particular instance of the dilute multiparticle system by ρ_ω_(**r**) and its scattered intensity by *I*_ω_(**q**), where ω stands for the orientation states of all contained *N*_p_ particles. In a typical FXS experiment (Fig. 1 ▸), the instantaneous scattered intensity 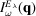 measured on a large-area detector represents a 2D cut of the 3D scattered intensity *I*_ω_(**q**) defined by a portion of the Ewald sphere *E*_λ_, where λ denotes the wavelength of the incident X-ray beam, indicating the dependence of the Ewald sphere radius on the photon energy. This geometric condition can be formulated in spherical coordinates (*q* ≥ 0, 0 ≤ θ ≤ π, 0 ≤ ϕ < 2π) as a *q* dependence of the polar angle θ (Saldin *et al.*, 2009 ▸), 

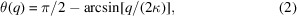

where κ = 2π/λ is the angular wavenumber, *q* = 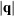 = 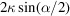 is the magnitude of the scattering vector, α is the scattering angle, and thus 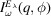 = 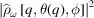 [see Fig. 1 ▸(*b*)]. By slight abuse of notation, we shall from now on use *I*_ω_ to denote both the full 3D intensity distribution of a sample and its 2D cut 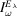 along the Ewald sphere.

The central idea of FXS is that from a collection of *M* scattering images 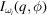 , *i* = 1, …, *M*, corresponding to *M* random realizations of the multiparticle system, information about the single-particle electron density ρ(**r**) [equation (1)] can be extracted. This can be accomplished using the angular CCFs (Kam, 1977 ▸, 1980 ▸). In the present study we employ the average two-point CCF defined at distinct momentum transfer magnitudes *q* and *q*′ as (Kam, 1977 ▸) 

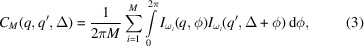

where 0 ≤ Δ < 2π is the angular coordinate and statistical averaging is performed over *M* scattering patterns.

### 2.2. Rotational invariants in fluctuation X-ray scattering

**Figure 2:**
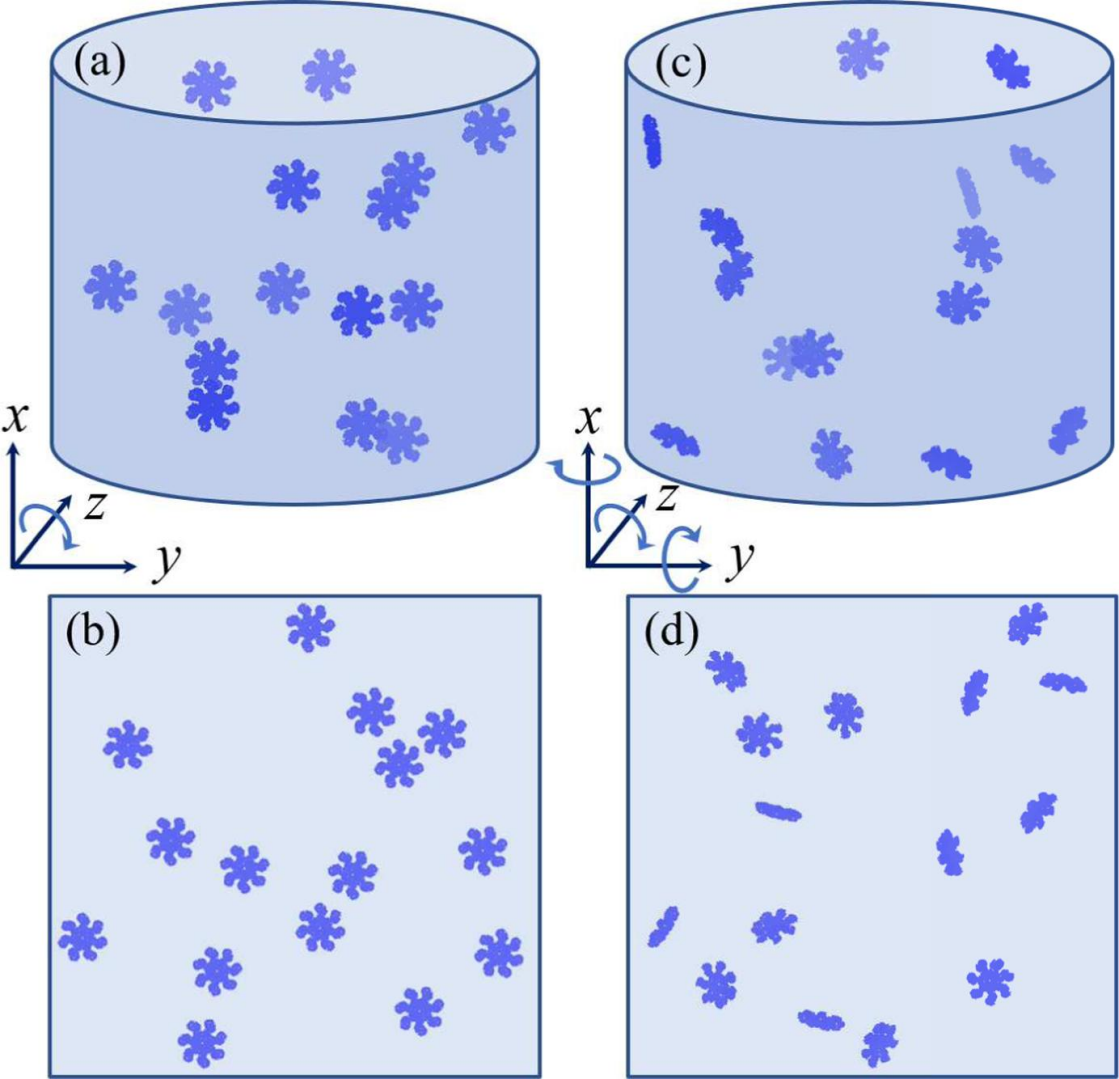
Snapshots of the samples corresponding to the (*a*), (*b*) 2D and (*c*), (*d*) 3D cases (see Section 2.2). Particle orientations are uniformly distributed (*a*), (*b*) over the rotation group SO(2) about the *z* axis (which is parallel to the incident X-ray beam direction) and (*c*), (*d*) over the rotation group SO(3). Here (*a*) and (*c*) are the bulk 3D samples, while (*b*) and (*d*) are the planar 2D samples.

In order to establish the connection between the single-particle intensity *I*(**q**) [equation (1)] and *C*_*M*_(*q*, *q*′, Δ) [equation (3)], it is customary to express *I*(**q**) using a suitable orthonormal basis. In this work we consider two cases of practical interest, which we shall call the 2D and 3D cases: they correspond to uniform distributions of particle orientations ω_*i*_ over (*a*) the rotation group SO(2) in two dimensions or (*b*) the rotation group SO(3) in three (see Fig. 2 ▸). Using circular harmonics in the 2D case and spherical harmonics in the 3D case, it is possible to show that *I*(**q**) and *C*_*M*_(*q*, *q*′, Δ) can be related via the rotational invariants *B*_*n*_ and *B*_*l*_, respectively (Kam, 1977 ▸; Saldin *et al.*, 2009 ▸; Altarelli *et al.*, 2010 ▸, 2012 ▸; Donatelli *et al.*, 2015 ▸; Kurta *et al.*, 2016 ▸).

#### 2.2.1. 2D case: rotational invariants *B*_*n*_

In the 2D case we are interested in a 2D projection of the 3D particle structure, under the constraint that orientations of the particles composing a dilute system can only differ from each other by rotations around axes parallel to the incident X-ray beam [Figs. 2 ▸(*a*) and 2 ▸(*b*)]. Experimentally such situations have been realized in a study of nanoparticles deposited on a membrane (Pedrini *et al.*, 2013 ▸). According to the projection-slice theorem, the 2D projection of the structure is related to the scattered intensity distribution measured in a plane that cuts reciprocal space orthogonal to the incident-beam direction and passes through the reciprocal-space origin. Such measurements can only be performed at ‘flat Ewald sphere’ conditions, *e.g.* in SAXS geometry, when θ(*q*) ≃ π/2 [see equation (2)].

Considering the 2D single-particle scattering intensity [equation (1)] in polar coordinates (*q*, ϕ), the circular harmonic expansion (Fourier series expansion) of *I*(*q*, ϕ) can be specified as

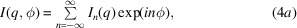




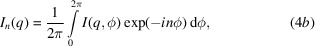

where *I*_*n*_(*q*) are circular harmonic expansion coefficients of the single-particle scattering intensity.

Using equation (4*a* [Disp-formula fd4a]) in (3), the average CCF at *M* → ∞ can be written as (Kurta *et al.*, 2013 ▸) 

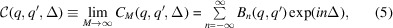

which identifies the invariants *B*_*n*_(*q*, *q*′) as circular harmonic expansion coefficients of 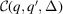 . Similar to the 3D case, this result is valid for a dilute system of particles (*N*_p_ ≥ 1), with *B*_*n*_(*q*, *q*′) expressed as 

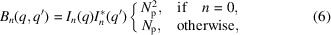

where the asterisk ‘*’ denotes complex conjugation.

Note that equation (6) provides a direct connection between the experimentally accessible invariants *B*_*n*_ and the harmonic expansion coefficients *I*_*n*_(*q*) of the 2D single-particle intensity. The rotational invariance of *B*_*n*_ is a direct consequence of the Fourier shift theorem. It implies that a rotation 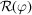 by an angle φ acts on the harmonic coefficients *I*_*n*_(*q*) by multiplication with a phase factor, *i.e.*


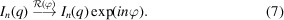




#### 2.2.2. 3D case: rotational invariants *B*_*l*_

In the 3D case we are interested in the 3D structure of particles, while the orientations of the particles composing a dilute system are uniformly distributed over SO(3) [Figs. 2 ▸(*c*) and 2 ▸(*d*)]. This situation corresponds to typical conditions in conventional biological SAXS measurements. The spherical harmonic expansion of the single-particle scattered intensity *I*(*q*, θ, ϕ) [equation (1)] can be specified as 

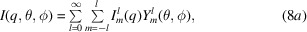




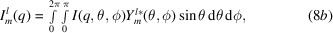

where 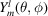 are spherical harmonics and 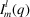 denote the expansion coefficients. By substituting equation (8*a* [Disp-formula fd8a]) into (3), the average CCF at *M* → ∞ can be expressed via (Kam, 1977 ▸; Saldin *et al.*, 2009 ▸) 

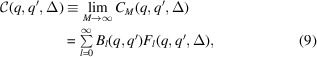

where *B*_*l*_(*q*, *q*′) represent the rotational invariants and *F*_*l*_(*q*, *q*′, Δ) is defined using Legendre polynomials *P*_*l*_ via 

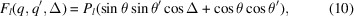

in which the angles θ and θ′ are related to *q* and *q*′ using equation (2). Importantly, equation (9) is also valid when averaging *C*_*M*_(*q*, *q*′, Δ) over scattering intensities 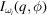 from a dilute system of (*N*_p_ ≥ 1) particles and the invariants *B*_*l*_(*q*, *q*′) can be expressed in terms of the expansion coefficients 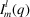 by 

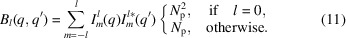




The rotational invariance of *B*_*l*_(*q*, *q*′) is a direct consequence of the fact that for a given order *l* the spherical harmonics 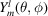 satisfy 

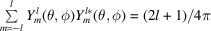

which, as a constant, is invariant under rotations. Alternatively, consider that a rotation 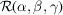 in SO(3) defined by Euler angles (α, β, γ) acts on the expansion coefficients 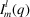 via the Wigner *D* matrices 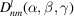 , *i.e.*


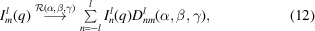

which satisfy the orthogonality condition 

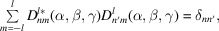

where 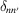 is the Kronecker delta (Rose, 1957 ▸).

### 2.3. Information content of *B*_*n*_ and *B*_*l*_

For simplicity, we shall proceed by considering the single-particle case (*N*_p_ = 1). Before making use of the invariants *B*_*n*_ and *B*_*l*_ [equations (6) and (11)] for single-particle structure recovery, it is instructive to understand how much information they retain about the single-particle intensity *I*(**q**). Considering a discretization of the momentum transfer variable *q* [see equation (47) in Appendix *A* [App appa]], it is possible to treat the harmonic coefficients as complex matrices, that is **I**_*n*_ for fixed *n* is a matrix of size *N* × 1 (*i.e.* a column vector) with elements *I*_*n*_(*q*_*k*_), and **I**_*l*_ for fixed *l* is a matrix of size *N* × (2*l* + 1) with elements 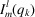 . This allows one to express the invariants for fixed orders *n* and *l* as matrix products,

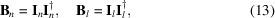

where the symbol ‘†’ denotes the conjugate transpose. By construction, these are positive semi-definite Hermitian matrices, and thus diagonalizable with positive eigenvalues (Saldin *et al.*, 2009 ▸; Donatelli *et al.*, 2015 ▸). The maximal rank of **B**_*n*_ is 1, while the maximal rank of **B**_*l*_ is *N*_*l*_ = min(2*l* + 1, *N*). Together this means that there exists a complex vector **v**_*n*_ of length *N* and a positive eigenvalue λ_*n*_, as well as a complex *N* × *N*_*l*_ matrix **V**_*l*_ and a diagonal matrix **Λ**_*l*_ of eigenvalues 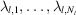 , such that 

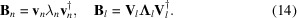

Equations (13) and (14) show two different decompositions of the same positive semi-definite matrices **B**_*n*_ and **B**_*l*_ in the 2D and 3D cases, respectively. Since such decompositions are unique up to unitary transformations (Kam, 1977 ▸), there exists a complex phase factor **u**_*n*_ such that **u** **u** ^†^ = 1 (here the phase factor is a complex number with an absolute value of 1 or, equivalently, a complex unitary matrix **u** of size 1 × 1, *i.e.* a singleton matrix), as well as a complex matrix **U**_*l*_ of size *N*_*l*_ × (2*l* + 1) such that 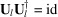 (hereafter ‘id’ stands for the identity matrix), which satisfy 

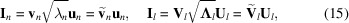

where for brevity we defined 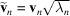 and 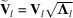 . One may notice the formal analogy of the expressions for **I**_*n*_ and **I**_*l*_. The information contained in each invariant is thus enough to specify the corresponding intensity harmonic coefficients, *I*_*n*_(*q*) or 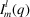 , up to a unitary matrix for each expansion order. In the present context, the problem of determining these unknown unitary matrices is analogous to solving the orientation determination problem in SPI.

In coherent X-ray diffraction imaging (CXDI), and particularly in SPI, to reconstruct the real-space structure one seeks to solve the phase problem using the measured scattering intensities as constraints (Chapman *et al.*, 2006 ▸). The inverse problem in FXS can be solved in a similar way, by employing the measured invariants *B*_*n*_ and *B*_*l*_ as constraints. The main approaches to solving the inverse problem in FXS include analytical phasing (Kurta *et al.*, 2013 ▸; Pedrini *et al.*, 2013 ▸), iterative phasing (Donatelli *et al.*, 2015 ▸; Kurta *et al.*, 2017 ▸; Pande *et al.*, 2018 ▸) and optimization (Saldin *et al.*, 2011 ▸; von Ardenne *et al.*, 2018 ▸). The single-particle structure reconstruction workflow presented below is based on the multitiered iterative phase retrieval algorithm (MTIP) (Donatelli *et al.*, 2015 ▸). The MTIP algorithm represents a generalization of conventional iterative phasing schemes employed in CXDI and enables *ab initio* 2D and 3D single-particle structure recovery (*e.g.* without symmetry constraints) from the rotational invariants *B*_*n*_ and *B*_*l*_.

## 3. Single-particle structure reconstruction workflow

A complete workflow for single-particle structure determination from diffraction patterns measured in an FXS experiment includes a number of procedures:

(i) Statistical averaging of the angular two-point CCF.

(ii) Extraction of rotational invariants (*B*_*n*_ or *B*_*l*_) from the CCF.

(iii) Reconstruction of the single-particle density ρ(**r**) and intensity *I*(**q**) via iterative phasing using the invariants.

(iv) Alignment and averaging of the reconstruction results.

A detailed description of the procedures implemented in our reconstruction workflow is provided in the following subsections.

### 3.1. Calculation of the average two-point CCF

In practical calculations of the angular CCF (3) we consider a uniform polar grid. The angular grid points, Δ_*t*_ and ϕ_*t*_, are given by Δ_*t*_ = ϕ_*t*_ = *t*2π/*N*_ϕ_, where *N*_ϕ_ is the number of angular grid points, and the radial sampling points are defined in equation (47). The average two-point CCF can be determined on this grid as 

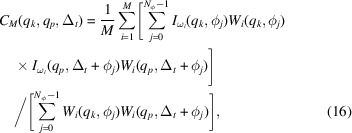

where *W*_*i*_(*q*_*k*_, ϕ_*j*_) is a binary mask for the *i*th image (Zaluzhnyy *et al.*, 2017 ▸). The mask has the value of 0 for all sampling points (*q*_*k*_, ϕ_*j*_) for which image data should be excluded (masked) from the analysis, and the value of 1 otherwise. Equation (16) suggests that, in practice, the CCF can be successfully determined even if many pixels are masked on individual diffraction patterns (see Section S4 of the supporting information), in the limiting case allowing measurements to be made using two-point detectors [see, for instance, Clark *et al.* (1983 ▸)].

### 3.2. Extraction of the rotational invariants

In the 2D case, equation (5) directly identifies the invariants *B*_*n*_(*q*, *q*′) as the coefficients of the circular harmonic expansion of the averaged CCF 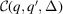 , and thus their determination is straightforward. In the 3D case, the relation between the invariants *B*_*l*_(*q*, *q*′) and 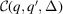 , given by equations (9) and (10), is more complicated. At flat Ewald sphere conditions (small-angle approximation) equation (10) simplifies to 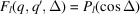 . In this case equation (9) represents the Legendre series expansion of 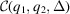 , and the invariants *B*_*l*_(*q*, *q*′) can be extracted by applying the inverse Legendre transform to 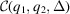 . In general, at curved Ewald sphere conditions [θ(*q*) ≠ π/2], the inverse Legendre transform extraction is not applicable since *F*_*l*_(*q*, *q*′, Δ) do not satisfy the orthogonality property of Legendre polynomials; therefore other approaches need to be applied.

A common way of extracting the invariants *B*_*l*_(*q*, *q*′) is to consider equation (9), for each pair of fixed *q* and *q*′ and discretized angular coordinate Δ [see *C*_*M*_(*q*_*k*_, *q*_*p*_, Δ_*t*_) in equation (16)], as a system of *N*_Δ_ linear equations. By treating the CCF 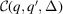 at fixed *q* and *q*′ as a vector 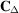 of size *N*_Δ_, the invariant *B*_*l*_(*q*, *q*′) as a vector **B**_*l*_ of size *L* + 1, and *F*_*l*_(*q*, *q*′, Δ) as an *N*_Δ_ × (*L* + 1) matrix 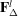 , it is possible to specify this system of linear equations as 

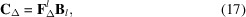

where *L* is the maximal considered invariant order, defined by the complexity of the particle structure and extent of the measured correlation data in reciprocal space. In practical applications, usually *N*_Δ_ > *L*; therefore, the matrix 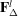 is not square, and the overdetermined system of linear equations (17) may only be solved approximately. Usually least-squares methods such as the pseudo-inversion (Ford, 2014 ▸), based on a singular value decomposition of 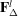 , are used to solve such linear systems.

Here we propose a different extraction method for the invariants **B**_*l*_ which is based on the circular harmonic expansion coefficients of the averaged CCF. It is possible to express *F*_*l*_(*q*, *q*′, Δ) in equation (9) in terms of spherical harmonics 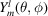 as (Saldin *et al.*, 2009 ▸) 

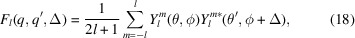

while the spherical harmonics can be specified using the associated Legendre polynomials 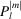 as 

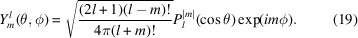

By combining equations (18) and (19) with (9), the averaged CCF can be expressed as 

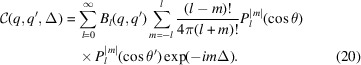

From this equation one may see that the circular harmonic expansion coefficients 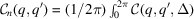 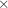 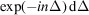 take the form 

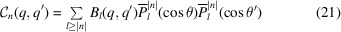

with 

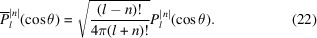

Since the associated Legendre polynomials 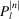 vanish for *l* < |*n*|, the summation in equation (21) involves only orders *l* ≥ |*n*|. This means that **B**_*l*_ for a given *l* is completely determined by the circular harmonic expansion coefficients 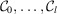 , up to the order *n* = *l*. Considering a cutoff order *L* we obtain a linear system of equations that can be written in matrix form as 

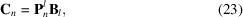

where *n*, *l* ≤ *L*, and 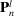 is the (*L* + 1) × (*L* + 1) upper-triangular matrix whose elements are 

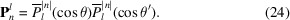

The upper-triangular linear system (23) can be directly solved using back-substitution (Ford, 2014 ▸). The computational complexity of the proposed method of extraction of **B**_*l*_, including calculation of the Fourier coefficients 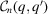 , is given by 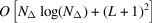 , as opposed to 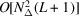 when solving equation (17) directly using singular value decomposition.

Note that *B*_0_(*q*, *q*′) can be directly determined from the measured SAXS intensity profiles, 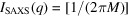 × 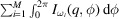 [see equation (3)], as *B*_0_(*q*, *q*′) = 4π*I*_SAXS_(*q*)*I*_SAXS_(*q*′), where the 4π factor is due to the chosen normalization of the spherical harmonics [see equation (19)]. This way of determining *B*_0_(*q*, *q*′) should be preferred in the case of noisy diffraction patterns, and is unavoidable if more complex forms of the CCFs are applied to mitigate background scattering [see, for instance, Kurta *et al.* (2017 ▸)].

### 3.3. Iterative phasing using the MTIP algorithm

For *ab initio* single-particle structure determination from the extracted invariants **B**_*n*_ or **B**_*l*_, we employ the MTIP algorithm (Donatelli *et al.*, 2015 ▸; Kommera *et al.*, 2021 ▸) with certain modifications (see Sections 3.3.3 and 3.3.4). Similarly to other iterative phasing methods used in CXDI/SPI, the single-particle structure is recovered by iteratively enforcing constraints in real and reciprocal spaces. Details of the implemented iterative phasing loop are described in the following subsections.

#### 3.3.1. Real-space constraints

A finite size of a particle leads to the formulation of the commonly used real-space support constraint, which defines a region of space where the electron density is expected to have nonzero values. Each reconstruction run starts with an initial random guess for the electron density ρ(**r**) of a particle. In our implementation the initial support function S(**r**) is defined as a sphere, which can be optimally set to the expected size of the reconstruction target. As the reconstruction progresses the support constraint is systematically updated according to the shrinkwrap (SW) algorithm (Marchesini *et al.*, 2003 ▸), that is the updated support is determined as an isosurface at a specified threshold of the convolution between the current density guess ρ′(**r**) and a Gaussian function, *i.e.*


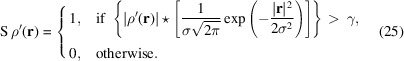

Here 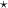 denotes convolution, and the free parameters σ and γ define the standard deviation of the Gaussian function and a relative threshold, respectively. Usually, the initial value of σ is chosen to be slightly above the expected full-period resolution of the reconstructed density and gradually decreases as the reconstruction progresses. The threshold γ is defined relative to the maximum density value 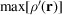 in the current phasing iteration and remains constant.

An often encountered set of real-space constraints used in X-ray imaging can be formulated as a (density) value projection *P*_V_, implemented here in the following general form: 

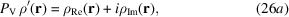

with 

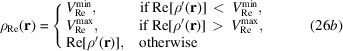

and 

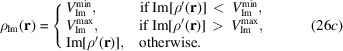

Re(·) and Im(·) define the real and imaginary parts of the corresponding arguments, and 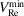 , 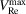 , 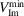 and 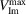 are free parameters. Depending on a particular choice of these four parameters, it is possible to set arbitrary bounds on the real and imaginary parts of ρ(**r**), particularly to impose reality or non-negativity. The value projection of ρ′(**r**) on the support function can then be defined as 

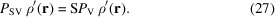

**Figure 3:**
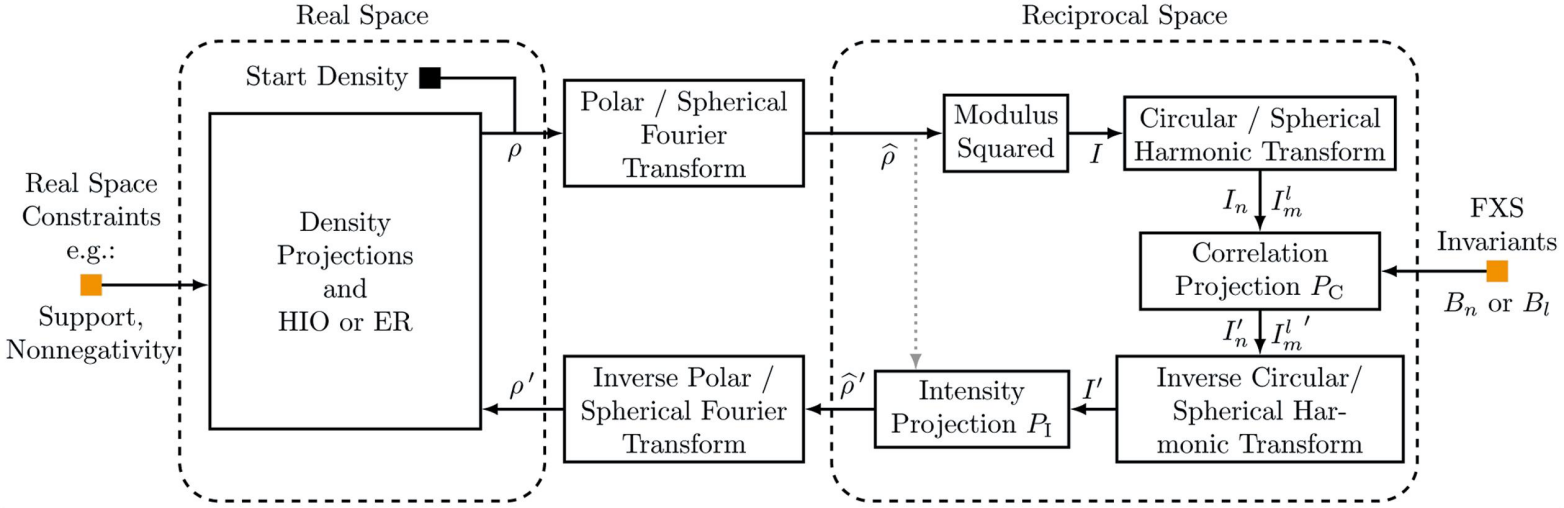
A scheme of the implemented MTIP loop. The filled orange squares mark the entry points for constraints, and the black square denotes the initial density guess. The quantities labeled on the scheme (ρ, *I*, *I_n_*/ 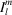 *etc*.) should be interpreted as iterative estimates of the corresponding theoretical quantities defined in equations (4)[Disp-formula fd4a] [Disp-formula fd4b]–(11).

The real-space domain incorporates the well known X-ray imaging algorithms, such as error reduction (ER) (Gerchberg & Saxton, 1972 ▸) and hybrid input–output (HIO) (Fienup, 1982 ▸). Iterative update of ρ(**r**) by these methods can be expressed using the projection *P*_SV_. In the ER scheme, the input electron density in the (*i* + 1)th iteration is defined using the output of the *i*th iteration as (see Fig. 3 ▸) 

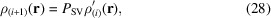

while for the HIO algorithm we have

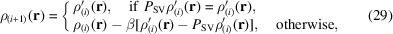

where β ∈ (0, 1] is a free parameter that regulates the strength of the negative feedback (Fienup, 1982 ▸; Donatelli *et al.*, 2015 ▸).

#### 3.3.2. Reciprocal-space constraints

The main reciprocal-space constraint is realized by means of the correlation projection *P*_C_ (see Fig. 3 ▸), which takes the current approximation of the single-particle intensity harmonic coefficients *I*_*n*_ or 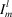 and determines the closest function, in the discrete *L* ^2^ norm, whose harmonic coefficients comply with equation (15).

In the 2D case this corresponds to finding the complex phase factors *u*_*n*_ (with |*u*_*n*_| = 1) for which the discrete *L* ^2^ distance between **I**_*n*_ and 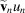 becomes minimal (Donatelli *et al.*, 2015 ▸), that is 

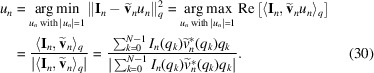

|| · ||_*q*_ and 〈 ·, · 〉_*q*_ are the *L* ^2^ norm and scalar product, respectively, weighted by *q*_*k*_ in order to comply with a continuous *L* ^2^ norm defined on a spherical grid. In the derivation of equation (30) it was considered that the maximal real part of the scalar product 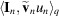 is obtained for the phase factor *u*_*n*_ that forces it to become real. Using the latter result, it is possible to formulate the correlation projection *P*_C_ in the 2D case as 

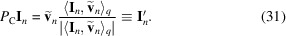

Analogously, in the 3D case one seeks to minimize (Donatelli *et al.*, 2015 ▸) 

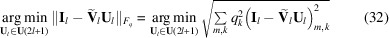

over all unitary matrices **U**_*l*_ of size 2*l* + 1, where 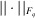 is the Frobenius norm (Ford, 2014 ▸) weighted by the square of the radial points 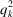 . The weighting factors 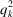 are again present to comply with the *L* ^2^ norm defined on a spherical grid. Such a minimization problem is known as a unitary Procrustes problem (Gower & Dijksterhuis, 2004 ▸). Instead of zero padding 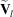 in the case of *N*_*l*_ ≤ (2*l* + 1), we alter the minimization constraint of equation (32) in requiring **U**_*l*_ to be a semi-unitary matrix, by which we mean an *N*_*l*_ × (2*l* + 1) matrix that satisfies 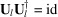 . A solution to the minimization problem (32) is found using the singular value decomposition 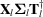 of the *N*_*l*_ × (2*l* + 1) matrix 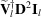 , where 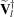 is the conjugate transpose of 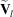 defined in equation (15), **D** is the *N* × *N* diagonal matrix of radial grid points **D** = diag(*q*_0_, …, *q*_*N*−1_), while 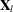 and 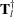 are unitary matrices of sizes *N*_*l*_ × *N*_*l*_ and *N*_*l*_ × (2*l* + 1), respectively, and 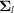 is an *N*_*l*_ × *N*_*l*_ diagonal matrix of non-negative singular values. The minimizing matrix **U**_*l*_ is then given by 

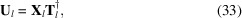

and consequently the correlation projection *P*_C_ in the 3D case can be specified as 

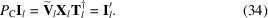




The other projection applied in reciprocal space is the intensity projection *P*_I_ defined as 

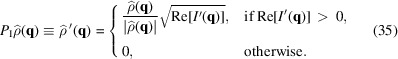

This is formulated similarly to the Fourier modulus projection, which serves as the main reciprocal-space constraint in conventional CXDI/SPI. Note that the described formalism differs from conventional SPI in that here *I*′(**q**) is not the experimentally measured scattered intensity but rather its current approximation, which is, along with the real-space density 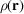 , iteratively refined using the measured invariants *B*_*n*_ and *B*_*l*_ as constraints.

#### 3.3.3. Polar and spherical Fourier transforms

Since the main reciprocal projection *P*_C_ is formulated in terms of harmonic coefficients of the scattered intensity, the need arises to implement the complete phasing loop (see Fig. 3 ▸) on a polar/spherical grid (including the Fourier transforms), in order to avoid the inaccuracies and performance limitations which would be imposed otherwise by repeated interpolations between Cartesian and polar/spherical grids. There is, however, no discrete Fourier transform in polar or spherical coordinates which would allow for repeated forward and inverse transforms. The approach applied here, as proposed by Donatelli *et al.* (2015 ▸), is to numerically approximate Hankel transforms, which connect the harmonic expansion of a function to the harmonic expansion of its Fourier transform. Consider ρ_*n*_(*r*) and 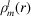 to be the harmonic expansion coefficients of an electron density in polar and spherical coordinates, and let 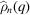 and 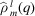 be the expansion coefficients of the respective scattering amplitudes (Fourier transformed densities). The connection between ρ_*n*_(*r*) and 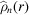 in the 2D case is then given by the Hankel transform 

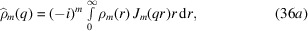




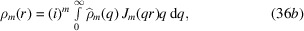

where *J*_*m*_ are Bessel functions of the first kind on integer order *m*. In the spherical (3D) case one finds

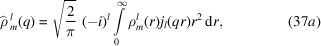




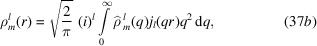

where *j*_*l*_ are spherical Bessel functions. One approach to numerically approximate the continuous Hankel transforms given in equations (36)[Disp-formula fd36a] [Disp-formula fd36b] and (37)[Disp-formula fd37a] [Disp-formula fd37b] is to expand the harmonic coefficients ρ_*m*_(*r*) or 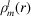 (and their reciprocal-space counterparts) in some orthogonal basis, thereby shifting the Hankel integral to the expansion functions (see Appendix *B* [App appb]). In the original version of MTIP this is accomplished using the cosine/sine series expansions (Donatelli *et al.*, 2015 ▸) (see Appendix *B*2). We also developed another approximation based on Zernike polynomial expansions, which allowed us to obtain closed-form expressions for the quadrature weights of the discretized Hankel transform (see Appendix *B*3). Further investigation, however, showed that both approaches converge to direct approximations of the integrals in equations (36)[Disp-formula fd36a] [Disp-formula fd36b] and (37)[Disp-formula fd37a] [Disp-formula fd37b] using a Riemann sum (see Appendices *B*1 and *B*4). We therefore employ the midpoint rule as a default approximation scheme for the Hankel integrals in our reconstruction workflow (see Appendix *B*4). In the 2D case, the Hankel transform (36)[Disp-formula fd36a] [Disp-formula fd36b] can thus be approximated on a discrete polar grid as 

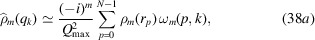




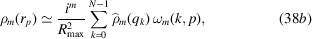

with the quadrature weights ω_*m*_(*p*, *k*) being defined by 

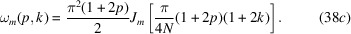

In the 3D case, the spherical Hankel transform (37)[Disp-formula fd37a] [Disp-formula fd37b] is approximated by 

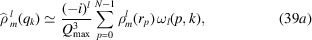




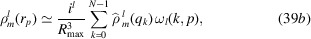

using the quadrature weights 

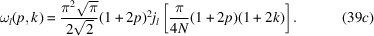

Note that the weights in the inverse transforms (38*b* [Disp-formula fd38b]) and (39*b* [Disp-formula fd39b]) are determined by transposing the parameters *p* and *k* in the weight functions specified for the forward transforms in equations (38*c* [Disp-formula fd38c]) and (39*c* [Disp-formula fd39c]), respectively.

#### 3.3.4. Fourier transform stabilization

We empirically found that stabilizing the Fourier transforms in the iterative loop by the following procedure may improve the convergence of reconstructions. The basic idea behind this operation is to reduce possible errors due to approximating the continuous Fourier transform in each MTIP iteration (see Section 3.3.3). Using the notation in Fig. 3 ▸ this correction can be expressed by modifying the definition of 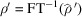 as 

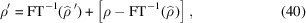

where FT^−1^ denotes the inverse Fourier transform. In the limit of a completely converged MTIP reconstruction, *i.e.* when 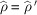 , meaning that the reciprocal-space projections do not change the intensity anymore, this definition ensures that the modified density ρ′ coincides with the input density ρ of the current iteration. Without this procedure ρ′ and ρ would differ due to the applied Fourier transform approximations.

#### 3.3.5. Error metrics

The evolution of the iterative phasing process can be tracked using several metrics, which may serve as convergence and error estimates. In analogy to error metrics commonly used in conventional X-ray imaging (Fienup, 1978 ▸, 1982 ▸), we define the relative normalized errors in reciprocal and real space as (see Fig. 3 ▸) 

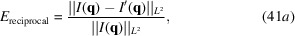




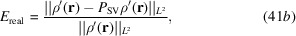

where *P*_SV_ is the density projection defined in equation (27). Here 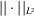 denotes the *L* ^2^ norm in polar/spherical coordinates, that is 

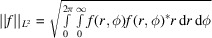

and 

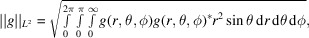

for square integrable functions *f*(*r*, ϕ) and *g*(*r*, θ, ϕ). Since, as previously mentioned, the single-particle scattered intensity is initially unknown in FXS and reconstructed during the phasing process, the metrics *E*_reciprocal_ and *E*_real_ can only serve as convergence indicators and do not directly estimate the deviation of the current solution from experimental observables. For this reason, we also define metrics for determining the relative difference in the *L* ^2^ norm on the level of the invariants as 

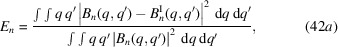




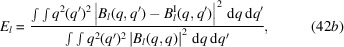

where *B*_*n*_(*q*, *q*′) and *B*_*l*_(*q*, *q*′) denote the input invariants employed as constraints, while 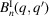 and 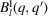 are the invariants calculated from the harmonic coefficients *I*_*n*_(*q*) and 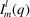 corresponding to the current phasing loop iteration (see Fig. 3 ▸).

### 3.4. Alignment and averaging of reconstructions

The invariants *B*_*n*_ or *B*_*l*_, employed as input data in the phasing process, do not contain (by definition) any information about the absolute position, orientation and point inversion of a particle in space. Therefore, individual reconstructions ρ(**r**) initiated from a random density guess may vary in these properties. Similarly to conventional iterative phasing schemes, the MTIP algorithm may also produce nonunique solutions (Donatelli *et al.*, 2015 ▸). Therefore, it is customary to present the final solution as an average of selected and aligned individual reconstructions.

Combining the action of rotations on the intensity harmonic coefficients in two [equation (7)] and three dimensions [equation (12)], in their matrix form, with equation (15) allows one to examine their action on the level of the unknowns **u**_*n*_ and **U**_*l*_ via 

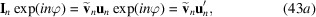




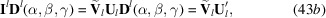

where we interpret **D** ^*l*^(α, β, γ) for each *l* as a (2*l* + 1) × (2*l* + 1) matrix. Since in the 2D case **u**_*n*_ is itself a phase factor, the rotational freedom in φ allows us to freely specify 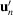 for a single chosen order *n* during the iterative phasing process. This condition causes the number of possible orientation states an individual reconstruction can attain to become finite. This, in turn, enables *a posteriori* algebraic orientation determination on the level of individual 2D reconstructions (see Section S2 of the supporting information).

In the 3D case, the restriction posed by equation (43*b* [Disp-formula fd43b]) is not strong enough to fix any of the unknown matrices 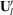 during the reconstruction process. Therefore, orientational alignment of 3D reconstructions is performed after completing the iterative phasing as follows. First, all reconstructions are centered at their respective centers of density and a reference reconstruction ρ_ref_ is selected. All reconstructions are then orientationally aligned with respect to this reference using fast Fourier transforms on the special orthogonal group SO(3) as described by Kostelec & Rockmore (2008 ▸). This procedure enables efficient calculations of the rotational cross-correlation 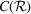 between the reference ρ_ref_(*r*, θ, ϕ) and any other reconstructed density ρ(*r*, θ, ϕ), which is given by 

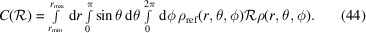




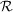
 is a rotation in SO(3) and 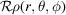 is a rotated version of the reconstructed density ρ(*r*, θ, ϕ).

The cross-correlation 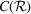 is maximal at the rotation 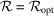 for which the rotated density 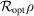 optimally matches the corresponding reference ρ_ref_. To facilitate structure alignment it is helpful to limit the range (*r*_min_, *r*_max_) to regions of the reconstructed densities that are not spherically symmetric. In order to correct for a possible point inversion in the reconstructions, we apply this alignment procedure to each 3D reconstruction ρ, as well as its point-inverse ρ_inv_, resulting in two aligned candidates per reconstruction. Finally, we determine the relative distance of the two candidates 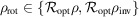 from the reference density ρ_ref_ using the *L* ^2^ norm, 

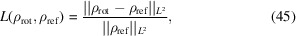

and select the candidate ρ_rot_ with the lowest distance for subsequent averaging.

Note that, in the 2D case, after centering and aligning the reconstructions according to the procedure described in Section S2 of the supporting information, we also use equation (45) to correct for point inversion.

The presented algorithm allows one to select the reconstructions to be used in the final average on the basis of their error metrics (41)[Disp-formula fd41a] [Disp-formula fd41b] and (42)[Disp-formula fd42a] [Disp-formula fd42b], as well as their distance (45) from the reference structure. Finally a resolution estimate of the average can be computed using a generalized version of the phase retrieval transfer function (PRTF) (Kurta *et al.*, 2017 ▸), 

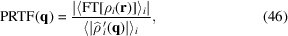

where 〈·〉_*i*_ denotes averaging over the selected collection of aligned reconstructions and FT[ρ_*i*_(**r**)] is the Fourier transform of the *i*th aligned electron density (see Fig. 3 ▸). If we assume that 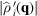 are identical in all individual reconstructions, as is the case in conventional CXDI [where 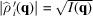 and *I*(**q**) is the experimentally determined intensity], expression (46) reduces to the conventional PRTF formula [see *e.g.* Chapman *et al.* (2006 ▸)].

## 4. *xFrame*: a Python implementation of the reconstruction workflow

The reconstruction workflow described in Section 3 is implemented in the open-source software suite *xFrame* available at https://github.com/European-XFEL/xFrame. The software consists of the back-end framework, which takes care of technical details unrelated to the reconstruction process (*e.g.* multiprocessing, GPU access, data storage, input settings *etc.*), and the *fxs* project which implements various routines of the reconstruction pipeline (calculations of the CCF, extraction of invariants, iterative phasing, averaging of reconstructions).

### 4.1. Dependencies

Table 1 ▸ lists *xFrame* dependencies and their usage. For computationally expensive operations such as the Fourier and harmonic transforms, we use existing software that references to C or Fortran routines wherever possible. In all other cases we rely on *numpy* vectorization and GPU acceleration using OpenCL. Although *xFrame* depends exclusively on cross-platform packages, it has currently only been tested on Linux-based operating systems.

### 4.2. Input/output data formats

*xFrame* requires input data in the form of a set of diffraction patterns in binary format or a statistically averaged two-point CCF *C*_*M*_(*q*, *q*′, Δ) in HDF5 format. Human-readable YAML files are used to specify the input settings for different *xFrame* routines. The output data produced by *xFrame* are stored in four standard formats, which are HDF5, YAML, VTK and PNG. The HDF5 format is used for general-purpose data storage, *e.g.* to save calculated metrics and reconstruction results, and the output YAML files are used to store the input settings associated with a particular reconstruction. Finally, VTK files and PNG images target visualization of reconstruction results. Specifically, the open-source VTK file format allows one to examine the reconstructed densities on their native spherical or polar grid without any further postprocessing.

### 4.3. *xFrame* usage

**Figure 4:**
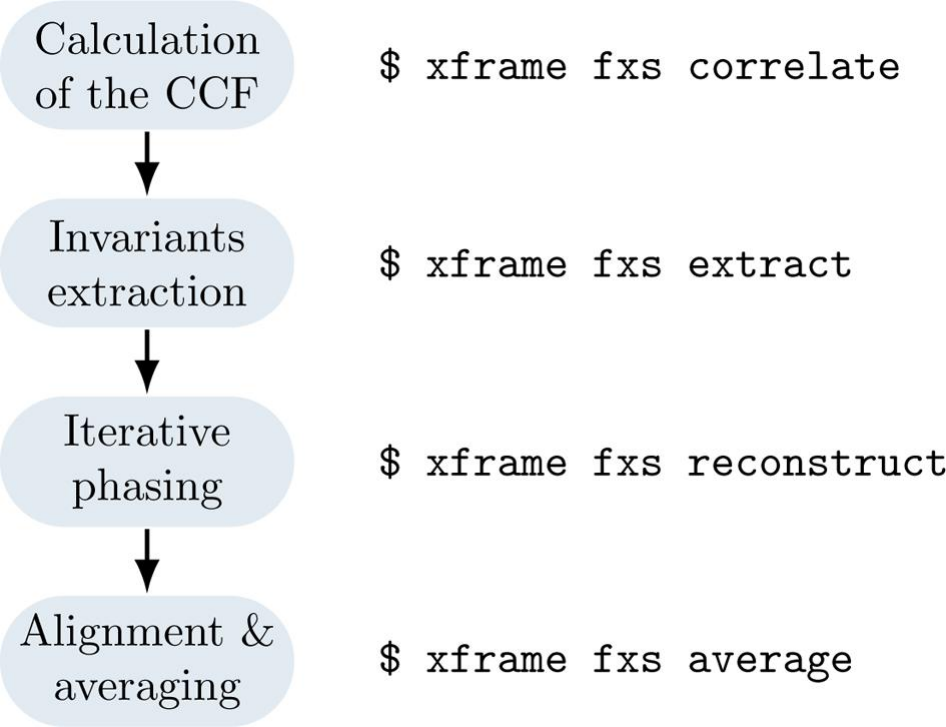
Diagram of a typical reconstruction workflow (left) using the command-line tools (right) of *xFrame*. Each of the *xFrame* commands takes as an argument a human-readable settings file that specifies all relevant options.

A typical reconstruction pipeline using the command-line interface of *xFrame* is shown in Fig. 4 ▸. It is possible to enter the workflow at different points, by running xframe fxs correlate to compute the CCF (16) from a set of input diffraction patterns, or extracting the rotational invariants *B*_*n*_ or *B*_*l*_ from the two-point CCF (by running xframe fxs extract with a specified input CCF in HDF5 format), or directly running reconstructions using the extracted invariants (xframe fxs reconstruct), which can then be aligned and averaged in a final step using xframe fxs average. Apart from the command-line interface it is also possible to use *xFrame* directly as a Python module. Details on the installation process as well as tutorials can be found at https://xframe-fxs.readthedocs.io.

The iterative phasing process is implemented in a way that allows one to run a single reconstruction per available CPU core using the Python *multiprocessing* module, while at the same time access to the GPU resources is shared among all parallel reconstructions (see Section S1 in the supporting information).

## 5. Reconstructions from simulated data using *xFrame*

**Figure 5:**
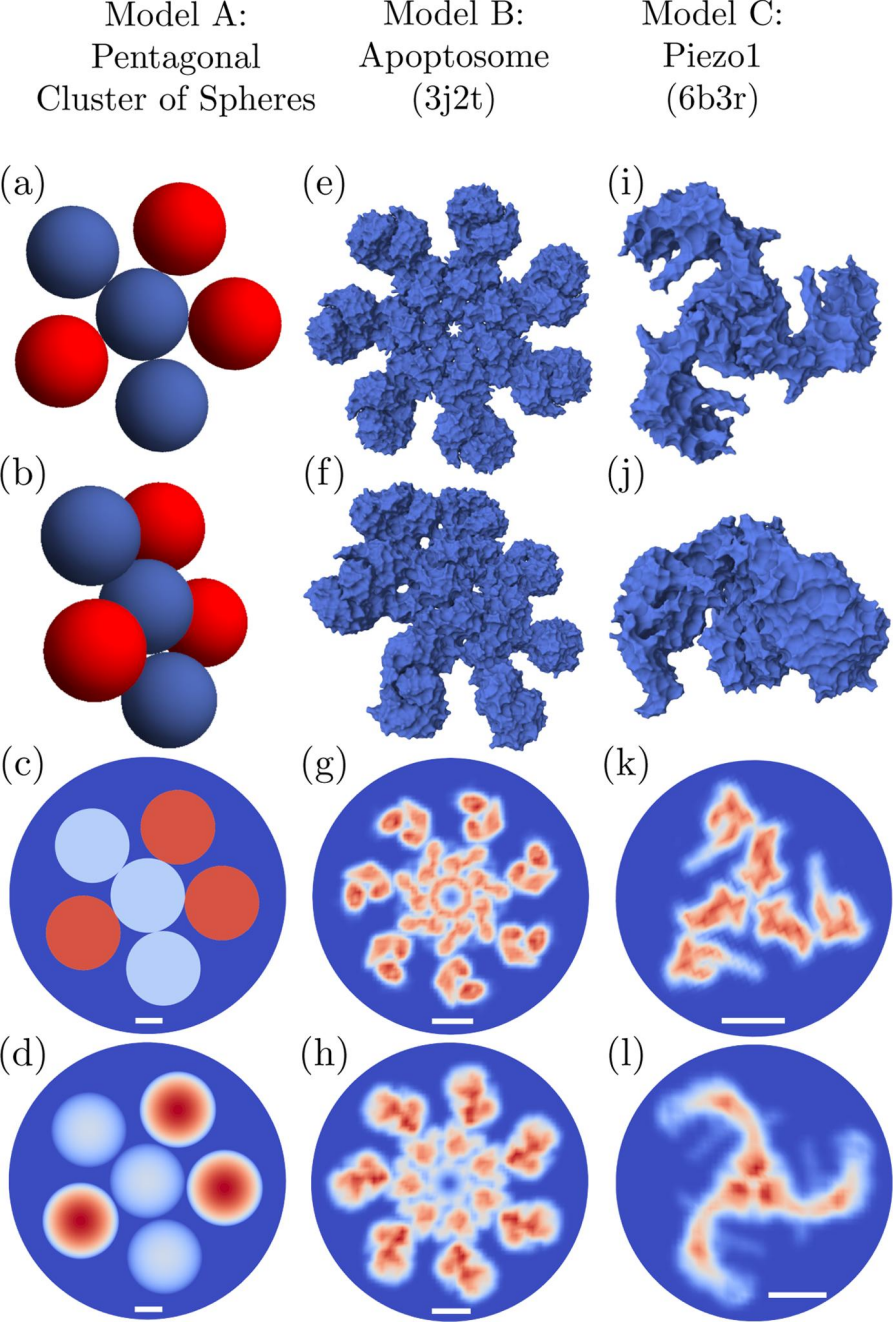
Three model structures (models A–C) considered for reconstructions using *xFrame*: (*a*)–(*d*) a pentagonal cluster consisting of spheres of uniform density with a diameter of 140 nm, with the red spheres being of doubled density as compared with the blue spheres; (*e*)–(*h*) the human apoptosome (PDB entry 3j2t; Yuan *et al.*, 2013 ▸); (*i*)–(*l*) the mechano­sensitive ion channel Piezo1 (PDB entry 6b3r; Guo & MacKinnon, 2017 ▸). The two upper rows show distinct views of the 3D structures. The third row displays 2D slices through the centers of the respective 3D models, and the bottom row displays 2D projections on the image plane, which were produced using the electron-density maps generated in *UCSF Chimera* (Pettersen *et al.*, 2004 ▸) for the corresponding 3D models. The white scale bars shown in the two bottom rows correspond to 5 nm.

Here we demonstrate single-particle structure recovery from simulated FXS data using *xFrame*. The scattering intensities (1) were simulated assuming ideal kinematic X-ray scattering without noise. We considered dilute limit approximation, where inter-particle interference can be neglected, and simulated 10^5^ diffraction patterns for each of the considered model structures presented in Fig. 5 ▸. Diffraction patterns were computed up to a maximum momentum transfer *Q*_max_ = 0.32 Å^−1^ for model A, and up to *Q*_max_ = 0.42 Å^−1^ for models B and C. The diffraction patterns were then used to determine the averaged CCF (16), and subsequently the invariants *B*_*n*_ and *B*_*l*_.

**Figure 6:**
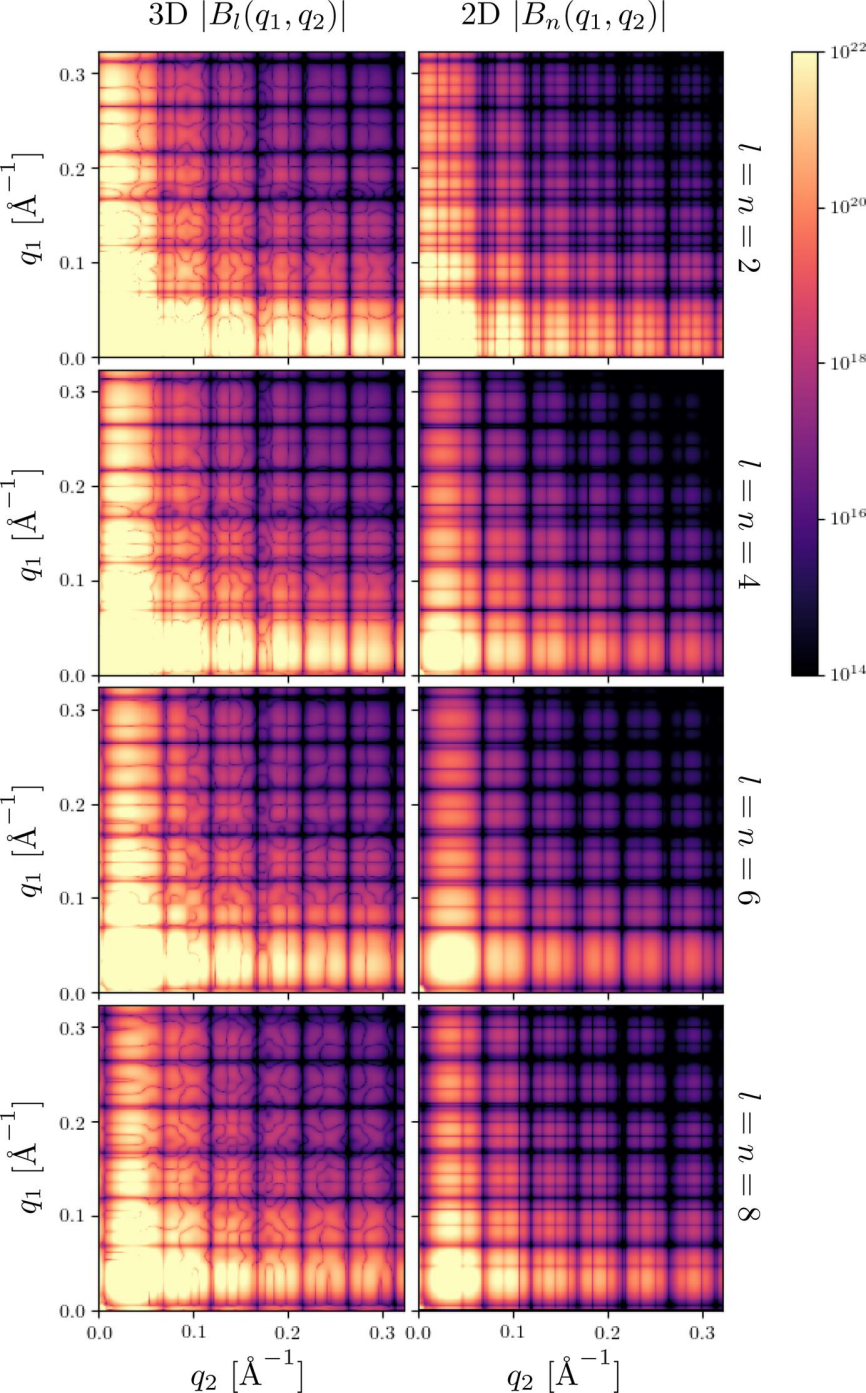
Absolute values of the rotational invariants 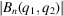 and 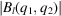 of orders *n*, *l* = 2, 4, 6 and 8, determined for model A in the 2D case (right) and 3D case (left). The invariants 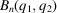 show features in the form of straight lines, which is a direct consequence of the fact that each 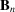 is a matrix of rank 1. The invariants 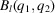 display more complex features since the respective matrix 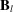 can have a rank higher than 1 (see Section 2.3).

We first consider the reconstruction results for the single-particle case (*N*_p_ = 1), where the input set of diffraction patterns was simulated from single particles in random orientations in two or three dimensions. For instance, the invariants extracted [by solving equation (23)] from the 2D and 3D FXS data sets simulated for a single pentagonal cluster of spheres (model A) are shown in Fig. 6 ▸.

**Figure 7:**
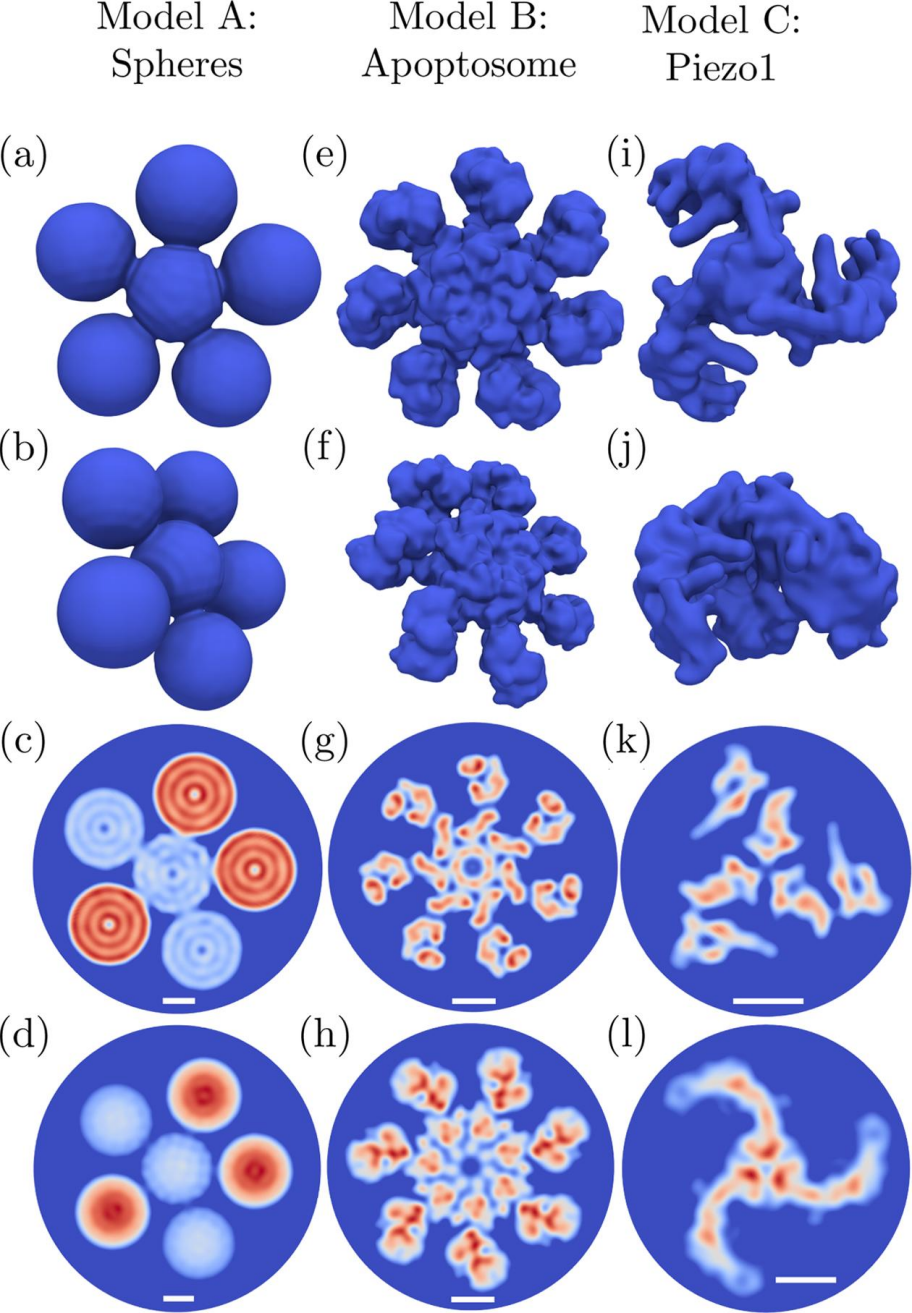
Averaged 2D and 3D reconstructions obtained using *xFrame* for three different model structures shown in Fig. 5 ▸. The two upper rows show two distinct views of the reconstructed 3D structures, and the third row displays 2D slices through the centers of the respective 3D reconstructions. The bottom row displays averaged 2D reconstructions, which correspond to the 2D projections shown in the bottom row in Fig. 5 ▸. The first two rows show isosurfaces at 15% of the maximal reconstructed electron-density value, and the last two rows display density values higher than 15% of the maximal density value. The 2D slices in (*c*), (*g*) and (*k*) are taken at approximately the same regions of the electron density as given for the model structures in (*c*), (*g*) and (*k*) of Fig. 5 ▸, respectively. The white scale bars shown in the two bottom rows correspond to 5 nm.

The complete iterative phasing process in *xFrame* is divided into the main and refinement stages, where the electron density with the lowest error metric obtained during the main phasing stage is further optimized in the refinement stage. *xFrame* allows one to separately set up the number, sequence and parameters of ER, HIO and SW procedures (see Section 3.3.1) in the main and refinement stages. For the 3D reconstructions shown in Fig. 7 ▸ the main stage consisted of blocks of 60× HIO, followed by 1× SW and 40× ER steps. The number of used iteration blocks varied from five in the case of model A to 30 for model B and model C. The 3D refinement part consisted of a single block of 1× SW followed by 200× ER steps for all considered models.

The obtained 2D reconstructions [Figs. 7 ▸(*d*), 7 ▸(*h*) and 7 ▸(*l*)] were produced using ten main stage iteration blocks consisting of 500× HIO followed by 1× SW and 200× ER steps, while the refinement part consisted of 1× SW step followed by 200× ER iterations. The HIO parameter β was determined in the *i*th iteration as β(*i*) = *a* exp(*bi*) + *c*, with parameters *a*, *b* and *c* chosen in such a way that β(*i*) was exponentially decreasing during the reconstruction process from 0.5 down to 0.14 for the 3D reconstructions, and from 0.1 down to 0.01 for the 2D reconstructions. The SW threshold value γ [equation (25)] was set to 0.11, and the standard deviation σ was linearly decreasing from 2 nm to 1.5 nm during the reconstruction for all models. The density value projection [equations (26)[Disp-formula fd26a] [Disp-formula fd26b] [Disp-formula fd26c]] was parameterized as 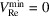 , 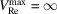 , 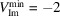 and 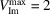 . We empirically found that allowing small nonzero values of 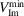 and 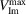 results in improved convergence rates of reconstructions. The Fourier transform stabilization has been applied as described in Section 3.3.4. The presented 3D reconstructions were obtained considering spherical harmonic expansion orders up to 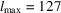 , while the 2D reconstructions used circular harmonic orders up to 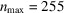 . The reciprocal projection used invariants *B*_*n*_ and *B*_*l*_ up to the same maximal orders while setting all odd-order invariants to 0. The considered number of radial steps was *N* = 256 for all models, and the angular sampling was chosen such that the maximal harmonic order could be resolved (see Appendix *A* [App appa]). To follow the reconstruction progress we used the error metric *E*_real_ defined in equation (41*b* [Disp-formula fd41b]).

**Figure 8:**
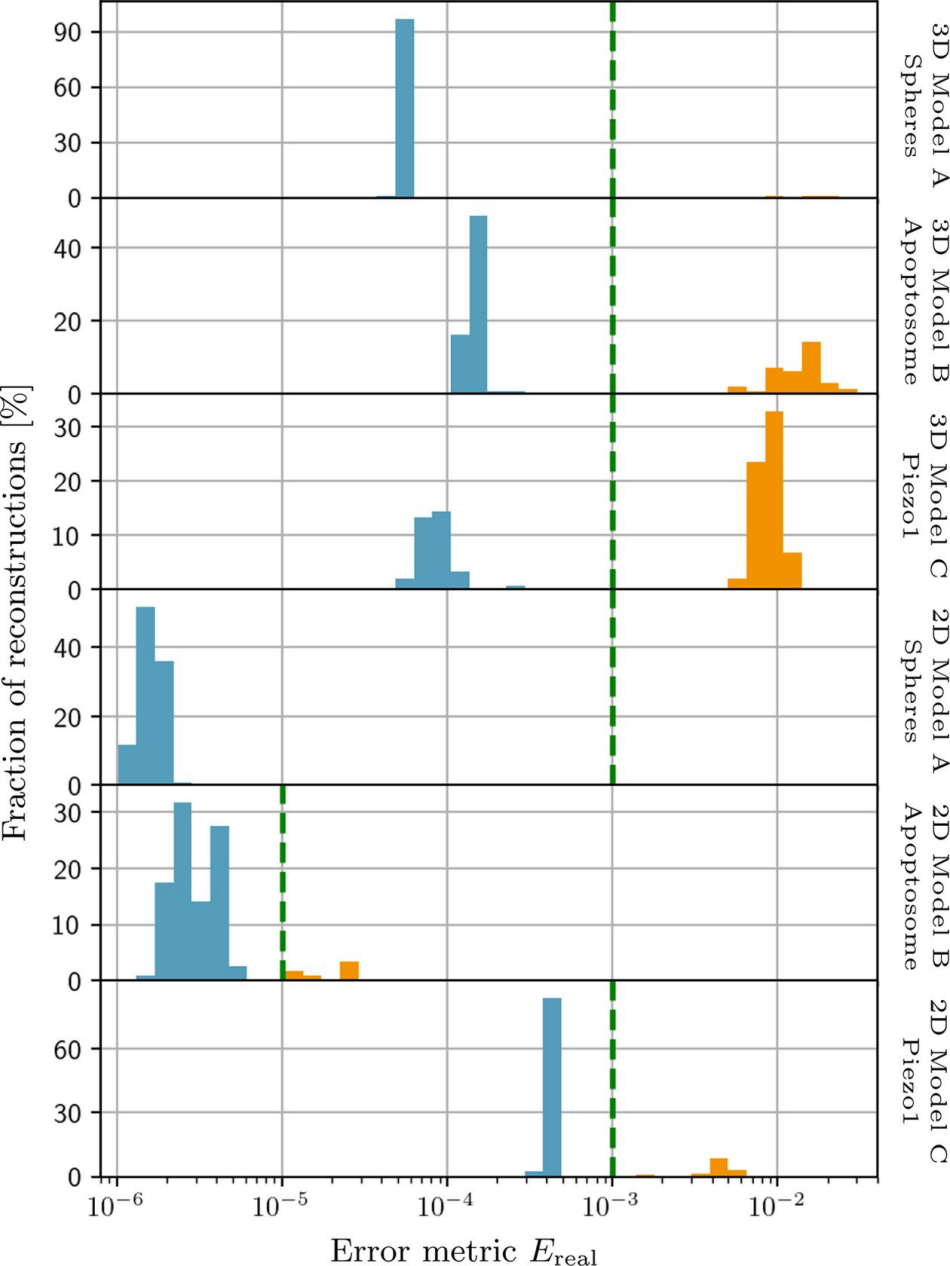
Normalized histograms of the final error metric values *E*_real_ [equation (41*b*)[Disp-formula fd41b]] shown for all individual 2D and 3D reconstructions listed in Table 2 ▸. For most of the models the reconstructions cluster into two groups separated by at least half an order of magnitude in their final error value, which allows one to identify converged reconstructions by introducing a threshold. The thresholds for each model are signified by the dashed green lines, placed at 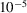 for the 2D model B, and at 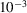 for all other models. Thus, the parts of the histograms shown in light-blue and orange colors correspond to converged and not converged reconstructions, respectively. For the 2D model A all computed reconstructions have approximately similar values of *E*_real_ and were considered to be converged. The 3D model A has a total of three reconstructions with error values around 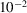 that did not converge.

**Figure 9:**
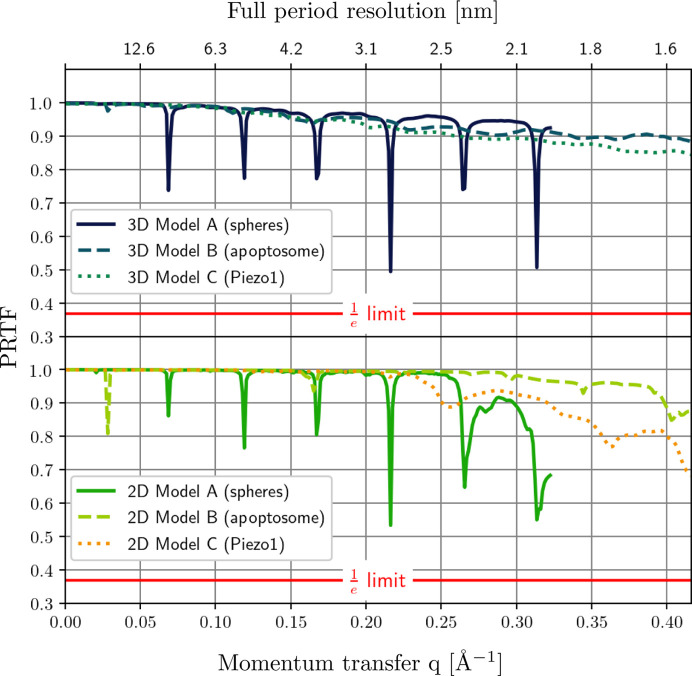
PRTF curves determined for the averaged 2D (bottom) and 3D (top) reconstructions shown in Fig. 7 ▸. The red lines define a cutoff value of 1/*e*, used to estimate the reconstruction resolution by PRTF. Since all PRTF curves are above this threshold, the resolution of the reconstructed structures is limited by the momentum transfer cutoffs in the respective input data, which are 0.32 Å^−1^ for model A and 0.42 Å^−1^ for models B and C.

Individual reconstructions were classified as ‘converged’ or ‘not converged’ according to the histograms of the final values of the error metric *E*_real_ (see Fig. 8 ▸ and Table 2 ▸). Note the different convergence rates for different structures in Table 2 ▸. The reconstructed structures shown in Fig. 7 ▸ were obtained by aligning and averaging 100 converged reconstructions for each model using xframe fxs average. The corresponding PRTF curves [equation (46)] computed for the single-particle reconstructions are shown in Fig. 9 ▸, indicating that the resolution is Fourier limited, *i.e.* limited by the extent of the simulated FXS data in reciprocal space. The impact of the maximum considered spherical harmonic expansion order 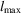 on the resolution of the obtained 3D reconstructions is illustrated in Fig. S4 of the supporting information.

The ability to perform X-ray scattering measurements from just individual particles (*N*_p_ = 1) in solution at near-physiological conditions represents an ideal scenario for FXS analysis, although it might be challenging to achieve in practice for weakly scattering bioparticles. The invariant-based FXS approach offers the possibility to perform reconstructions based on multiparticle (*N*_p_ > 1) X-ray scattering (see Sections 2.1 and 2.2), in which the total scattering from bioparticles is enhanced compared with single-particle measurements. According to equations (6) and (11), a scaled version of the single-particle invariants can be extracted from such multiparticle FXS data. Under the assumption that *N*_p_ is known, the invariants extracted from the multiparticle scattering data can be normalized, *i.e.* the zero harmonic order by 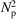 and all higher orders by *N*_p_, and used to perform single-particle structure recovery as described above. Reconstruction results for the multiparticle scattering case (*N*_p_ = 10) presented in Fig. S5 of the supporting information look very similar to those obtained in the single-particle case (Fig. 7 ▸). The sensitivity of the reconstruction results to the accuracy of the determined scaling factors *N*_p_ is demonstrated in Fig. S6 of the supporting information.

## 6. Summary and conclusions

In this work, we presented a workflow for single-particle structure determination from FXS measurements. The workflow consists of several steps, including calculation of the average two-point CCF from a set of diffraction patterns, extraction of rotational invariants from the CCF, iterative phasing of rotational invariants using the MTIP algorithm, and selection, alignment and averaging of individual reconstructions. We proposed a new method for extracting rotational invariants from the angular Fourier spectra of the CCF (see Section 3.2). We also introduced several modifications to the original version of the MTIP algorithm published by Donatelli *et al.* (2015 ▸), including discrete versions of the Hankel transform (Section 3.3.3) and additional measures to improve phasing stability (Sections 3.3.4 and 3.4).

We considered different approximations of the Hankel transform (see Appendix *B* [App appb]) using orthogonal basis expansions, including formulations based on the cosine/sine series expansion (Appendix *B*2) and Zernike polynomial expansion (Appendix *B*3), as well as direct approximation of Hankel integrals with Riemann sums using the midpoint rule (Appendix *B*4). As a byproduct, we derived a closed-form expression for the Hankel transform of the radial part of the 3D Zernike polynomials (Appendix *B*5). Our results show that the Hankel transform, defined via orthogonal basis expansion of density, produces results that converge to the results of direct approximation of the continuous Hankel integral with a Riemann sum (Appendix *B*1).

The proposed workflow has been implemented in the open-source software suite *xFrame*. *xFrame* features a multiprocessing scheme that allows for parallel reconstruction runs on a multi-core CPU while at the same time enabling GPU acceleration of time-consuming steps within each of the parallel reconstructions (see Section S1 in the supporting information). Successful reconstructions can be identified using distinct error metrics (Section 3.3.5), and subsequently aligned and averaged (Section 3.4). In the 2D case, the alignment routine is partially incorporated into the MTIP loop and completed *a posteriori* (see Section 3.4 and Section S2 of the supporting information). We demonstrated the functionality of *xFrame* by performing 2D and 3D reconstructions from simulated single-particle scattering data for several structures (Section 5). The results show successful *ab initio* recovery of the particle shapes and internal density distribution without the need to apply symmetry constraints. Reconstructions from multiparticle scattering data are also possible, while the feasibility of such reconstructions relies on accurate knowledge of the number of particles 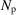 contributing to X-ray snapshots (see Section S3 of the supporting information).

FXS was originally proposed for single-particle structure determination from multiparticle solution X-ray scattering; thus, it complements traditional SAXS and SPI techniques. The inverse problem in FXS is solved using iterative phase retrieval, in which two phase problems are tackled simultaneously. The first involves finding unknown unitary matrices to determine the single-particle scattered intensity, which is equivalent to solving the orientation determination problem in conventional SPI. The second is related to finding optimal phases of the single-particle scattering amplitudes, similar to conventional iterative phase retrieval used in SPI or CXDI. At the same time, FXS may naturally expand the information content of traditional solution SAXS experiments if X-ray scattering measurements are performed on timescales faster than the rotation diffusion time of particles in solution. Such multiparticle measurements, however, require very precise detector corrections to be able to detect weak intensity fluctuations about orientationally averaged SAXS (see Section S4 of the supporting information for a brief summary of challenges related to experimental measurements and data processing). If such requirements can be fulfilled, FXS may potentially close the gap between conventional SPI, SAXS and crystallographic structure determination, particularly in time-resolved studies with an XFEL (Kurta *et al.*, 2023 ▸).

Although the single-particle structure reconstruction workflow is presented here in the context of biological applications, it may also serve as an alternative way for 2D and 3D structure determination of arbitrary molecules, nanoparticles, engineered nanostructures *etc.*, provided that FXS data of sufficient quality can be measured. We hope that the presented open-source software *xFrame* can facilitate efforts in this direction.

## Supplementary Material

Supporting text and illustrations. DOI: 10.1107/S1600576724000992/yr5118sup1.pdf

## Figures and Tables

**Table 1:** List of dependencies of *xFrame*

Package	Usage
*numpy* (Harris *et al.*, 2020 ▸)	All parts of *xFrame*
*scipy* (Virtanen *et al.*, 2020 ▸)	Invariants extraction
*pyOpenCL*	GPU access
*shtns* (Schaeffer, 2013 ▸)	Spherical harmonic transforms
*pysofft* (SOFT) (Kostelec & Rockmore, 2008 ▸)	3D alignment of reconstructions
*matplotlib* (Hunter, 2007 ▸)	2D plots
*openCV* (Bradski, 2000 ▸)	2D plots
*vtk* (Schroeder *et al.*, 2006 ▸)	3D plots
*h5py* (The HDF Group *et al.*, 2020 ▸)	Data storage
*ruamel.yaml*	Software settings
*click*	Command-line interface
*psutil*	Hardware information

**Table 2:** Reconstruction statistics using *xFrame*

Model	Reconstructions performed	Reconstructions converged (% of total)
3D model A	113	110 (97)
3D model B	168	111 (66)
3D model C	340	116 (34)
2D model A	120	120 (100)
2D model B	120	113 (94)
2D model C	120	103 (86)

## Appendix A Discrete polar and spherical grids

In practical applications data acquisition and processing are realized on a finite discrete grid, and hence our reconstruction workflow is implemented on a discrete polar/spherical grid. We define the extent and sampling of the radial coordinates in real and reciprocal space as

where the sets *r*_*p*_ and *q*_*k*_ of *N* radial points sample real and reciprocal space (in spherical or polar coordinates) up to a maximum extent *R*_max_ and *Q*_max_, respectively. In practice, *Q*_max_ is usually determined by the maximum extent of the measured scattered intensity on a detector (or quality of the CCF), while the real-space cutoff *R*_max_ can be justified by the finite dimensions of the particle, so that the particle density centered at the origin of the coordinate system satisfies the condition ρ(*R*_max_) = 0. The chosen discretization is compatible with the midpoint rule applied in this work for approximating integrals with Riemann sums (Hughes-Hallett *et al.*, 2012 ▸). We use the reciprocity relation *R*_max_ *Q*_max_ = π*N* in computations of the discrete Fourier transforms.

As for the angular coordinates, in the 2D polar grid we consider the angle ϕ to be uniformly sampled from 0 to 2π. In the case of the 3D spherical grid, this also applies to the azimuthal angle ϕ, while the polar angle θ is sampled from 0 to π on Gauss–Legendre nodes, *i.e.*  , where  is the *i*th zero of the Legendre polynomial of the order  , and  is the maximal spherical harmonic order considered in the expansion (8*a* [Disp-formula fd8a]).

## Appendix B Hankel transform approximations

In this appendix we consider different methods for approximating the continuous Hankel transforms in equations (36)[Disp-formula fd36a] [Disp-formula fd36b] and (37)[Disp-formula fd37a] [Disp-formula fd37b].

### b1. Expansion via orthogonal polynomials

One of the approaches to derive a discrete version of the Hankel transforms in equations (36)[Disp-formula fd36a] [Disp-formula fd36b] and (37)[Disp-formula fd37a] [Disp-formula fd37b] is to expand ρ_*m*_(*r*) or  using an orthogonal basis ξ_*i*_(*r*) of all square integrable functions on the interval [0, *R*_max_], *e.g.* in the 2D case

where ρ_*m*,*i*_ are the expansion coefficients. This allows one to shift the Hankel integration from ρ_*m*_(*r*) to the expansion functions ξ_*i*_(*r*), *i.e.* using equation (48*b* [Disp-formula fd48b]) in (36*a* [Disp-formula fd36a]) we get

The integral in equation (49) can be precomputed once independently of the considered function ρ_*m*_(*r*), and then used for all subsequent Hankel transform computations. The remaining integral in (48*a* [Disp-formula fd48a]) in the determination of the expansion coefficients ρ_*m*,*i*_ can then be approximated by using one of the available methods, *e.g.* the trapezoidal rule (Donatelli *et al.*, 2015 ▸) or midpoint rule (present work), to derive a discrete form of equations (36)[Disp-formula fd36a] [Disp-formula fd36b] and (37)[Disp-formula fd37a] [Disp-formula fd37b]. In the present case, equation (49) can be approximated in the following general form,

where *A*_*m*_ are constants, ω_*m*_(*k*, *q*) are quadrature weights defined by the integral in equation (49), and *r*_*p*_ and *q*_*k*_ are discrete coordinates in real and reciprocal space [see equation (47)]. Such an approach has been implemented by Donatelli *et al.* (2015 ▸) using a cosine/sine series expansion. An example of the cosine/sine approach, using the midpoint rule to approximate the expansion coefficients, is presented in Appendix *B*2. In this work we also developed an approximation of the Hankel transforms by employing Zernike polynomials as the orthogonal basis functions ξ_*i*_(*r*). The advantage of the obtained expressions is that, in this case, the integral in (49) can be evaluated analytically (see Appendix *B*3) and thus does not require numerical approximation as in the case of a cosine/sine series expansion (see Appendix *B*2).

At the same time, analysis of expression (49) defined in terms of an arbitrary orthogonal basis ξ_*i*_(*r*) reveals further aspects of such an expansion. By applying the midpoint rule to approximate the integral in (48*b* [Disp-formula fd48b]) on a discrete grid defined in Appendix *A* [App appa], and substituting the result into (49), we obtain

where  . Considering that  is a real function, the integral in (51) defines, in fact, the complex conjugated coefficients  of expansion of  in the basis ξ_*i*_(*r*), that is [see equation (48*a* [Disp-formula fd48a])]

Using the latter result in (51) and rearranging the terms we get

One may recognize that the expression in square brackets is exactly the series expansion of  in terms of ξ_*i*_(*r*_*p*_) [see equation (48*b* [Disp-formula fd48b])]. In the limit of infinite expansion orders (*i* → ∞) such an approximation scheme is, therefore, independent of the chosen orthogonal basis ξ_*i*_ and results in the following expression:

Note that equation (54) corresponds exactly to simple numeric evaluation of the continuous Hankel transform using a Riemann sum in the integration bounds [0, *R*_max_] [see equation (69) in Appendix *B*4]. Similar derivations can be performed for the inverse Hankel transform, and also in the 3D case. This conclusion is supported numerically for the 3D case in Fig. S7 of the supporting information, where it is shown that the Zernike [equation (68)] and cosine/sine [equations (56[Disp-formula fd56a] [Disp-formula fd56b])] weights approach the weights obtained by directly approximating the Hankel integrals using the midpoint rule [equation (39*c* [Disp-formula fd39c])]. In our reconstruction workflow, we therefore employ the quadrature weights from the midpoint formulation (see Appendix *B*4) as a default option, while the Zernike approximation is available for testing.

### b2. Cosine/sine series expansion approximation

The quadrature weights given by Donatelli *et al.* (2015 ▸) are determined by employing a cosine/sine series expansion of ρ_*m*_(*r*) or  , and their reciprocal-space counterparts. Using the definitions in Appendix *A* [App appa] and applying the midpoint rule for approximating the integrals, the weights for the 2D case take the following form for odd orders *m*,

and for even orders *m*,

where *s*_max_ defines the maximum expansion order in the cosine/sine series, *c*_*s*_ = 1/2 for *s* = 0, and *c*_*s*_ = 1 otherwise.

In the 3D case the weights for odd orders *l* are

and for even orders *l*,

The quadrature weights (55[Disp-formula fd55a] [Disp-formula fd55b]) and (56[Disp-formula fd56a] [Disp-formula fd56b]) can be directly used in forward transforms (38*a* [Disp-formula fd38a]) and (39*a* [Disp-formula fd39a]), respectively, while for the inverse transforms (38*b* [Disp-formula fd38b]) and (39*b* [Disp-formula fd39b]) the corresponding weight functions should be transposed with respect to *p* and *k*.

### b3. Zernike polynomial expansion approximation

Here we derive the quadrature weights ω_*m*_(*p*, *k*) and ω_*l*_(*p*, *k*) by employing the radial parts of the Zernike polynomials (Zernike, 1934 ▸; Born & Wolf, 2019 ▸) as the basis functions ξ_*i*_(*r*) in the expansion (48[Disp-formula fd48a] [Disp-formula fd48b]).

The radial parts  of the *D*-dimensional Zernike polynomials (Zernike & Brinkman, 1935 ▸) can be defined using Jacobi polynomials  as

for even *s* − *h*, and  otherwise, where *s* and *h* are non-negative integers.

The radial polynomials  form a set of orthogonal polynomials on the interval [0, 1], with the orthogonality condition given for an arbitrary *h* by

This implies that any sufficiently smooth function *f*(*r*) that is defined on a finite interval [0, *R*_max_] has a series expansion in the polynomials  , *i.e.*

The advantage of Zernike expansions is that the Hankel transform of  can be evaluated exactly. In the 2D case (*D* = 2) for *h* = *m* one finds

whereas in the 3D case (*D* = 3) for *h* = *l* we have

for *q* ≠ 0. A proof of equation (60) can be found in Appendix VII of Born & Wolf (2019 ▸), and equation (61) is derived in this work in Appendix *B*5[App appb]. Since we are interested in approximating the Hankel transforms of a function defined on a finite interval [0, *R*_max_], for an arbitrary positive *R*_max_, we use scaled versions of the integrals (60) and (61),

and

Let us now consider the Hankel transform for the 2D case, as specified in equation (49), and obtain its discrete version in the form of equation (50). By considering the expansion (59[Disp-formula fd59a] [Disp-formula fd59b]) of *f*(*r*) = ρ_*m*_(*r*) up to the maximum order *s* = *s*_max_, with each expansion coefficient ρ_*m*,*s*_ approximated using the midpoint rule, we can write

Since ρ_*m*_(*r*) is finitely supported, the integration range reduces from [0, ∞) to [0, *R*_max_]. Note that equation (64) is already represented as a weighted sum with weights that are independent of the function ρ_*m*_(*r*). Using the integral relation from equation (62), we arrive at (for *q* ≠ 0)

Considering the definitions in equation (47), we finally obtain the discrete version of the Hankel transform in the 2D case in the form of (50),

with the quadrature weights determined as

Following a similar procedure, the Zernike weights for the 3D case can be specified as

The quadrature weights (67) and (68) can be directly used in the forward transforms (38*a* [Disp-formula fd38a]) and (39*a* [Disp-formula fd39a]), respectively, while for the inverse transforms (38*b* [Disp-formula fd38b]) and (39*b* [Disp-formula fd39b]) the corresponding weights are obtained by transposing ω_*m*_(*p*, *k*) and ω_*l*_(*p*, *k*) with respect to *p* and *k*.

### b4. Direct approximation of the Hankel integrals using the midpoint rule

Here we consider direct approximations of the Hankel transforms given in equations (36[Disp-formula fd36a] [Disp-formula fd36b]) and (37[Disp-formula fd37a] [Disp-formula fd37b]) with Riemann sums.

In the 2D case the integral in (36*a* [Disp-formula fd36a]) can be approximated using the midpoint rule as

Using the definitions in Appendix *A* [App appa], we can present equation (69) in the form

where  and the quadrature weights ω_*m*_(*p*, *k*) are given in equation (38*c* [Disp-formula fd38c]).

Similarly, in the 3D case the integral in (37*a* [Disp-formula fd37a]) can be approximated as

where  and the quadrature weights ω_*l*_(*p*, *k*) are given in equation (39*c* [Disp-formula fd39c]). Approximations for the inverse Hankel transforms (36*b* [Disp-formula fd36b]) and (37*b* [Disp-formula fd37b]) can be obtained in a similar way, producing the discrete forward and inverse Hankel transforms given in equations (38[Disp-formula fd38a] [Disp-formula fd38b]) and (39[Disp-formula fd39a] [Disp-formula fd39b]), respectively.

### b5. Hankel transform of the radial part of the 3D Zernike polynomial

Here we provide a proof of equation (61) for the 3D Zernike polynomials, which closely follows the derivation of equation (60) given by Born & Wolf (2019 ▸). We shall need the Rodrigues’ formula for Jacobi polynomials  and the series expansion of spherical Bessel functions *j*_*l*_(*r*), which can be obtained from 10.2.2, 10.47.3 and 18.5(ii) of NIST (2023 ▸):

where  denotes the gamma function and *n*! is the factorial of a non-negative integer number *n*.

By substituting *z* = 1 − 2*r* ^2^ in equation (73) and considering in equation (57) Jacobi polynomials for β = 0, the radial part of the Zernike polynomials for *D* = 3 yields

with α = *l* + 1/2 and *n* = (*s* − *l*)/2. By expressing the spherical Bessel function in equation (72) in terms of the argument *z* = *qr*, and using equations (72) and (74) in the left-hand side of equation (61), we find the following expansion for the integral:

with

The integral in (76) can be reformulated by introducing the variable *u* = *r* ^2^, which yields

We shall now perform integration by parts in equation (77) for two cases, *p* ≥ *n* and *p* < *n*. Notice that a single application of integration by parts results in

taking into account that *n* − 1 < *n* and α > 0.

For *p* ≥ *n* we can then perform integration by parts *n* times in (77), which results in

One may recognize the remaining integral as the beta function [see 5.12.1 in NIST (2023 ▸)], which finally yields for *p* ≥ *n*

In the case of *p* < *n*, it is possible to perform integration by parts only *p* times in (77), which gives

Using equations (80) and (81) in (75), the latter can be rewritten as

where in the last step we considered that  and introduced a variable *k* = *p* − *n*. Note that the sum in equation (82), including the prefactor  , is precisely the series expansion of a spherical Bessel function *j*_*s*+1_(*q*) of the order *s* + 1 [see equation (72)]. This finally yields

which completes the proof of equation (61).
