# Supplementary material for: A workflow for single-particle structure determination via iterative phasing of rotational invariants in fluctuation X-ray scattering
Source: J Appl Crystallogr. 2024 Mar 15;57(Pt 2):324–43. doi: 10.1107/S1600576724000992 (PMC11001396; doi:10.1107/S1600576724000992)
Supplement: Supplementary file 1 [file j-57-00324-sup1.pdf]

JOURNAL OF
APPLIED
CRYSTALLOGRAPHY

Volume 57 (2024)

Supporting information for article:

A workflow for single-particle structure determination via iterative phasing of rotational invariants in fluctuation X-ray scattering

Tim B. Berberich, Serguei L. Molodtsov and Ruslan P. Kurta

S1. Multiprocessing scheme and reconstruction performance

The phasing process used in *xframe fxs reconstruct* is implemented such that it can be run on a single CPU core. This allows for simple parallelization by running several reconstructions in parallel if multiple CPU cores are available. An illustration of the the multiprocessing scheme including GPU acceleration is shown in Fig. S1. Notably, access to the GPUs is entirely handled by separate GPU workers, this allows one to limit the GPU memory requirements and thus enables performant phasing on larger grid sizes and harmonic order limits. When GPU acceleration is used the parallelism of individual phasing workers is slightly broken, since individual calls to a single graphics card have to happen sequentially. This causes the phasing processes to compete for GPU time. Since, however, the individual GPU workloads are quite small compared to the rest of the phasing loop, we found this effect to be negligible, as it can be seen in the performance Fig. S2.

xFrame currently handles multiprocessing using the python *multiprocessing* module. To access the graphics cards we chose to use *OpenCL* in order to be independent on graphics card manufacturers and make the software available on as many machines as possible.

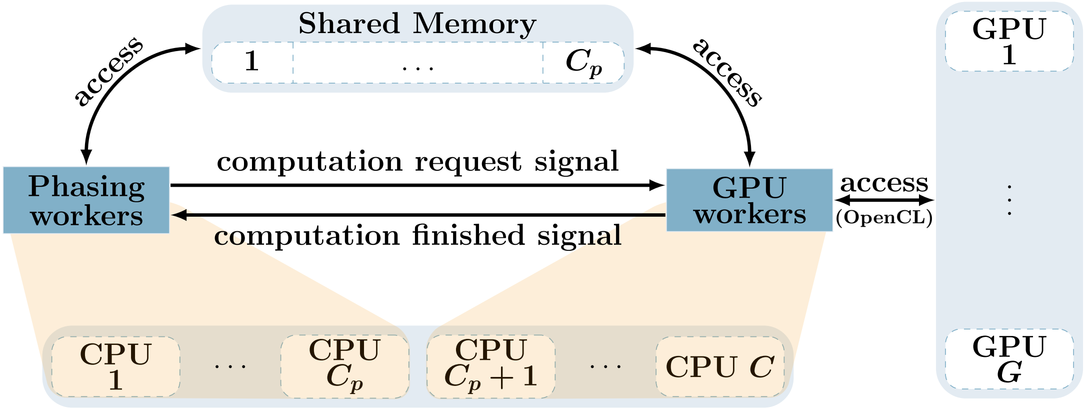

Fig. S1. During iterative phasing (see Fig. 3) the available CPU threads are divided into phasing and GPU workers. Each phasing worker is running an individual reconstruction, while a smaller number of GPU workers are accepting requests from the phasing workers to perform parts of the MTIP loop (Hankel transforms) on the available GPUs. The phasing and GPU workers communicate via simple Boolean signals, while the data transfer is handled indirectly via shared memory.

The following performance statistics where calculated on a single node running two AMD EPYC 7543 processors with a total of 64 physical CPU cores that access 512GB of RAM and two Nvidia RTX A6000 graphics cards with 48GB of memory each. Fig. S2 shows a comparison between computation times for three-dimensional MTIP reconstruction using 15 iterations of (60× HIO, 1 × SW, 40× ER) followed by a refinement stage of 200× ER. In these reconstructions 70 spherical harmonic orders with a constant angular sampling of 70 polar and 140 azimutal grid points were used while the number or radial grid points was varied from 64 to 256 with a step size of 16. Finally the error metric computed in each loop iteration is the one defined as E_{real} in equation (41b).

As can be seen in Fig. S2(a) the run-time of the presented algorithm depends linearly on the number of radial grid points, which is in agreement with the fact that all individual algorithm parts, except for the Hankel transforms, depend at most linear on the radial grid size. This is a good indication that no computational bottlenecks in memory or compute units where reached for the specified parameter ranges.

Furthermore, we observe an approximately constant multiprocessing speedup of around 28 times, which corresponds to roughly 50% of the theoretically attainable speedup, that is equal to the number of reconstructions executed in parallel (57 in the given example). These speedups show that the multiprocessing scheme depicted in Fig. S1 works as intended and the forced sequential access to individual GPUs is not breaking the CPU parallelization significantly. GPUs are currently exclusively used to compute the Hankel transforms (38a-39c), since their calculation would otherwise dominate the computation time. Moreover, the number of required computation steps for the Hankel transform depends quadratically on the number of radial grid points N. The overall linearity in increase of the computation time is a good indication that, within the tested radial grid sizes, the GPUs are able to compute each Hankel transform simultaneously at all considered radial grid points.

The relative time fraction a phasing loop spends on GPU operations, i.e. Hankel transforms, can be seen in Fig. S2(b) and is for all radial grid sizes smaller than 8%. This low value poses a future upgrade path for the presented algorithm in which also the harmonic transform calculations, which currently are the most time consuming operations, could be performed on GPUs.

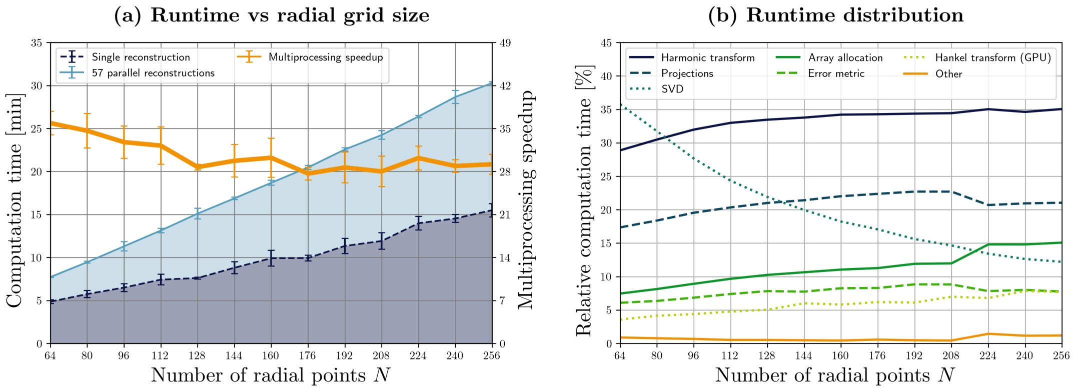

Fig. S2. Phasing performance of *xFrame* for 3D reconstructions of Model A as a function of the radial grid size N. (a) Phasing runtime and multiprocessing speedup. The runtime for a single reconstruction t_1 is compared to the total runtime t_{57} for 57 reconstructions running in parallel. The multiprocessing speedup is determined as $57 \cdot t_1/t_{57}$. The results were averaged over 10 independent runs for single and parallel reconstructions, with the depicted error bars indicating the standard deviations in t_1 and t_{57}. (b) Average runtime distribution among different types of computations involved in the phasing loop are illustrated for one of the 57 reconstruction processes running in parallel. Most of the phasing time is spent in the categories *Harmonic transform* (spherical harmonic transforms), *SVD* [solving the Procrustes problem in equation (32)], and *Projections* (reciprocal and real-space projections, including HIO, ER and SW), and less in *Array allocation* (numpy methods *array* or *copy*), *Error metric* [calculating E_{real}, see equation (41b)], *Hankel transform (GPU)* [Hankel transforms (39) implemented on GPUs] and *Other* (computation time that is not associated with any other category above).

S2. Alignment routine for 2D reconstructions

In Section 3.4 in the main text we noted that the overall orientational freedom of a particle allows us to freely specify, at reconstruction stage, an unknown phase factor u_{n_0} for a single chosen harmonic order n_0. In doing so, the corresponding expansion coefficient $I_{n_0}(q)$ of the single-particle intensity becomes completely defined. This causes the space of possible rotation states of any reconstruction to become finite, since only those rotations remain allowed that leave $I_{n_0}(q)$ unchanged. Consequently, the number of possible values for the remaining unknown phase factors u_n also becomes finite. After completing a particular reconstruction the determined values of u_n

can be modified by several rotation operations that bring each reconstruction into a common (reference) rotation state. Below we describe such an alignment algorithm.

In equations (7) and (43a) we noted how a rotation by an angle φ in SO(2) acts on the intensity harmonic coefficients $I_n(q)$, and the experimentally accessible quantities $\widetilde{\mathrm{v}}_n(q)$. Using this rotation action and demanding the invariance of u_{n_0} results in

$$u_{n_0} e^{i n_0 \varphi_j} = u_{n_0}, \quad \varphi_j = j\frac{2\pi}{n_0}, \quad j = 0, \ldots, n_0 - 1. \tag{S1}$$

Thus, there are n_0 distinct rotation angles φ_j that leave u_{n_0} invariant upon the rotation action. Since all of these rotations are integer multiples of φ_1, they lead to n_0 possible global rotation states attainable by each individual reconstruction. The task here is to bring all individual reconstructions to the same global rotation state with matching phase factors u_n.

Given any other harmonic order $n_1 \neq n_0$, we can apply one of the rotations φ_j that transform $\arg(u_{n_1})$ to some unique value, while leaving u_{n_0} unchanged. For example, we may request $\arg(u_{n_1})$ to take the minimum possible value after wrapping into the interval $(0, 2\pi)$, that is

$$\underset{\varphi \in \{\varphi_j\}}{\arg\min} \Big(\mathrm{mod}[\arg(u_{n_1}) + n_1 \varphi, 2\pi] \Big), \tag{S2}$$

and apply any of the rotations φ_j that solve the minimization problem (S2). In equation (S2) "mod" stands for the modulo operation used for phase wrapping.

After aligning the phase for the harmonic order n_1, only those rotations states φ_j remain possible, which leave both u_{n_0} and u_{n_1} invariant under rotation. The invariance condition for order n_1 takes a form similar to equation (S1), that is

$$u_{n_1} e^{i n_1 \varphi_k} = u_{n_1}, \quad \varphi_k = k\frac{2\pi}{n_1}, \quad k = 0, \ldots, n_1 - 1. \tag{S3}$$

Clearly, only those rotations φ_j leave both u_{n_0} and u_{n_1} invariant, which are present in both sets of rotations, $\{\varphi_j\}$ and $\{\varphi_k\}$, defined in equations (S1) and (S3), correspondingly. The set A of such rotations can be determined as a result of intersection

of the two sets of rotations, e.g. $A = \{\varphi_j\} \cup \{\varphi_k\}$. The number of rotations g_1 in the set A is equal to the greatest common divisor of n_1 and n_0, i.e. $g_1 = \gcd(n_1, n_0)$. These g_1 rotation states can be considered in the following steps to align the remaining harmonic orders. Notice, that if (in the example above) n_1 would be a multiple of n_0, then $g_1 = n_0$, and there are no rotation states in the set $\{\varphi_j\}$ that may alter $\arg(u_{n_1})$. Hence, all orders n that are multiples of n_0 may be excluded from the alignment procedure, since the corresponding arguments $\arg(u_n)$ cannot be further altered.

This allows us to define an algebraic alignment procedure, in which we successively choose harmonic orders n, and use the remaining rotational states to project $\arg(u_n)$ to its lowest possible value. The complete alignment algorithm can be formulated in steps as follows (see an example of its application in Fig. S3 for $n_0 = 12,\ n_1 = 8$, and $n_2 = 6$):

1. (Before reconstruction process) Define a sorted set of harmonic orders $O = \{n_t\}$, with $t \leq t_{\max}$, where $t_{\max}$ is the total number of harmonic coefficients considered in the reconstructions. Set $u_{n_0} = 1$ during the iterative phasing.

2. (After completing the reconstruction) In the 0-th alignment iteration ($i = 0$), compose a set A with possible global rotation states $\{\varphi_j\}$, where φ_j are defined in equation (S1), and set $g_0 = n_0$.

3. Remove all multiples of g_i from the set O. If O is empty (or $g_i = 2$) the alignment is finished, otherwise start the next iteration ($i \to i + 1$) in the next step.

4. Choose n_i to be the first remaining element of O. Choose one of the rotations φ_j from the set A that solves the minimization problem (S2) for n_i, and apply this rotation to all harmonic orders n_t present in the current set O, so that the updated phases are determined as $\arg(u_{n_t}) = \mathrm{mod}[\arg(u_{n_t}) + n_t\varphi_j, 2\pi]$.

5. Compose a set B with rotation states $\{\varphi_k\}$ determined for the harmonic order

n_i according to equation (S3).

6. Update the set of remaining free rotations A by intersecting it with the set B, that is $A = A \cup B = \{\varphi_j\} \cup \{\varphi_k\}$. The updated set A contains $g_i = \gcd(n_i, g_{i-1})$ rotation angles. Go to step 3.

Requiring the removal of all multiples of g_i (step 3) removes all orders whose phase factors can not be changed by the remaining rotations in A. Stated differently, this condition ensures that $g_i < g_{i-1}$, which means that the number of free rotations decreases after each alignment iteration. This causes the algorithm to stop after a finite (and typically small) number of iterations.

For robust performance of the algorithm it is important to sort the orders n_t in the set O according to the magnitude of the harmonic coefficients $|I_{n_t}|$ so that the most significant orders correspond to low indices t. To achieve this we ordered the intensity harmonic coefficients in descending order of their L^2-norms determined as

$$||I_n|| = \sqrt{\sum_k |I_n(q_k)|^2 q_k} = \sqrt{\sum_k |\widetilde{\mathrm{v}}_n(q_k)|^2 q_k}, \qquad \text{(S4)}$$

where $\widetilde{\mathrm{v}}_n(q_k)$ are the elements of the projection matrices $\widetilde{\mathbf{v}}_n$ introduced in equation (15). The matrices $\widetilde{\mathbf{v}}_n$ for small momentum transfer values q_k tend to be noisy, therefore it might be appropriate to exclude the low-q area from the summation in equation (S4).

After completing the alignment process, individual aligned reconstructions are unique up to point inversion (provided Friedel's law is satisfied). This final ambiguity is resolved using equation (45) as described in Section 3.4 in the main text.

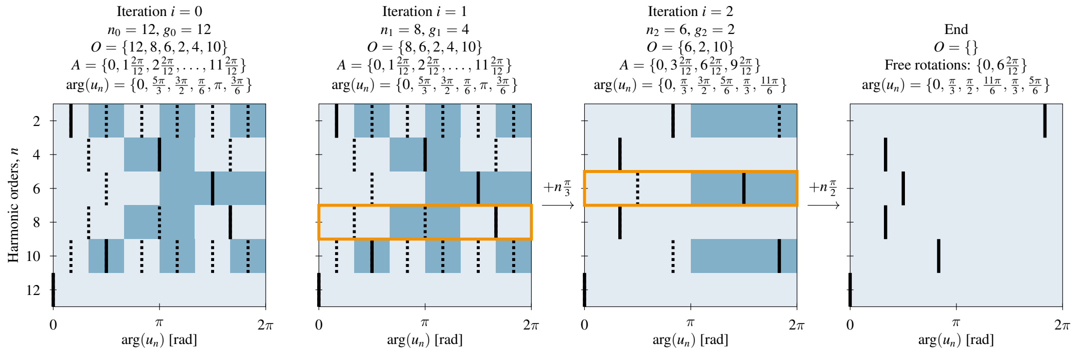

Fig. S3. Illustration of the alignment algorithm, where the specified values of parameters correspond to the beginning of each alignment iteration. The values of phases $\arg(u_n)$ (corresponding to the beginning of each alignment iteration) are specified for the respective orders n provided in the original set O at $i = 0$. They are shown in the plots as solid black vertical lines, while the dashed lines signify all other possible values permitted by the set of rotations A in a particular iteration. The orange rectangles highlight the harmonic order whose phase is constrained in a particular iteration. The left most figure displays iteration $i = 0$ directly after the reconstruction, in which we enforced $u_{12} = 1$ for $n_0 = 12$. In iteration $i = 1$ we identified, for harmonic order $n_1 = 8$, the rotation $\frac{\pi}{3}$ in the set A as the one producing the minimal phase of u_8, that is $\arg(u_8) = \text{mod}[\frac{5}{3}\pi + 8\frac{1}{3}\pi, 2\pi] = \frac{\pi}{3}$. This rotation is then applied to all orders present in the list O for the current iteration. The set A is then reduced according to step 6 of the algorithm, so that the updated set A contains only $g_1 = 4$ rotation angles at the beginning of iteration $i = 2$. We then fix the phase of $n_2 = 6$, and since $g_2 = 2$ the alignment process is completed after rotating the phases of the remaining harmonic orders in O by $\frac{\pi}{2}$. The final phases $\arg(u_n)$ for the aligned reconstruction are displayed in the right most figure.

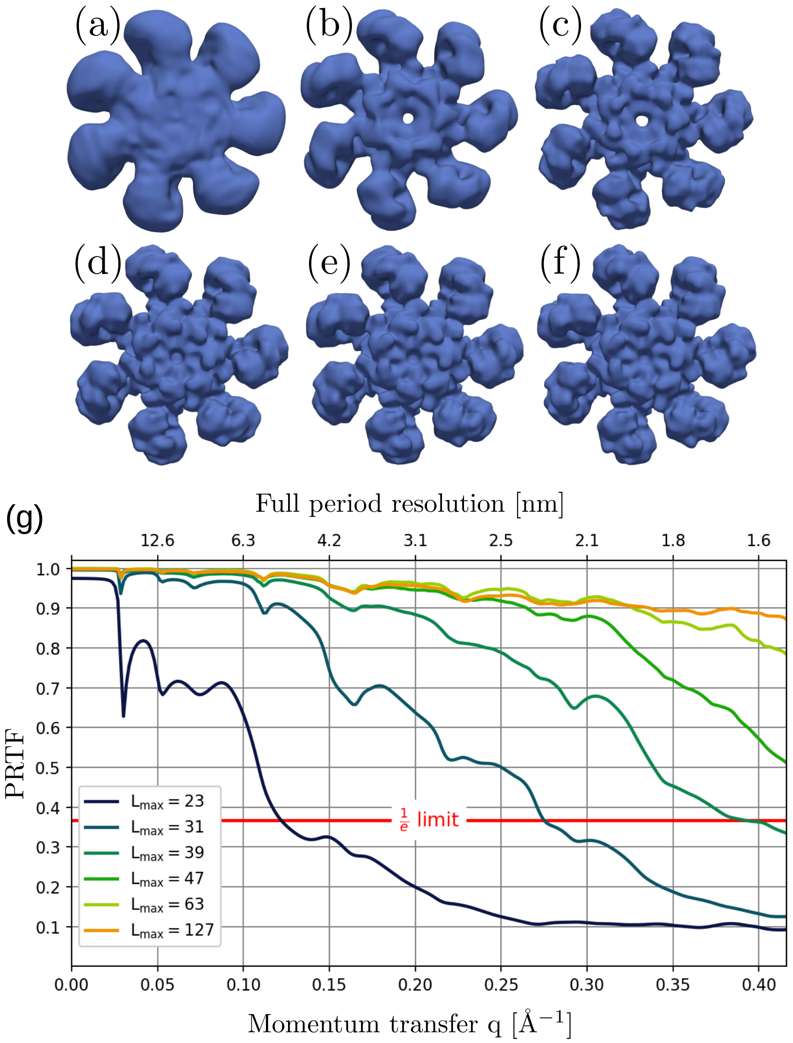

Fig. S4. (a)-(f) Averaged 3D reconstructions of Model B (human apoptosome) determined from single-particle scattering data for $l_{max} = 23, 31, 39, 47, 63$ and 127, correspondingly. Isosurfaces are taken at 15% of the maximal density of the respective reconstructions. (g) PRTF curves corresponding to the reconstructions in (a)-(f), showing a gradual decrease of resolution when restricting l_{max} to lower orders.

S3. Reconstructions from multiple-particle FXS data

To test *xFrame* performance on a multiple-particle scattering dataset, we used a stack of 10^5 simulated single-particle diffraction patterns and computed a set of 10^5 incoherently summed multi-particle patters, where each multiple-particle scattering pattern

is formed by randomly selecting and summing subsets of 10 single-particle patterns. By normalizing the extracted invariants according to equations (6) and (11) with $N_\mathrm{p} = 10$, and performing reconstructions using settings as described in Section 5 of the main text we obtained the averaged reconstructions shown in Fig. S5. As one can see, the results look very similar to those obtained for single-particle case (compare with Fig. 7 in the main text).

Reconstructions obtained from the multiple-particle FXS data are very sensitive to the relative scaling of the zero-order (B_0) and higher order invariants, that is, to the assumed number of particles N_p. As it is demonstrated in Fig. S6, for deviations of about 20% from the correct value for N_p, the averaged reconstructions still display most pronounced features of the particle shape, while the internal density distribution notably deviates from the expected one (see Fig. 5 in the main text).

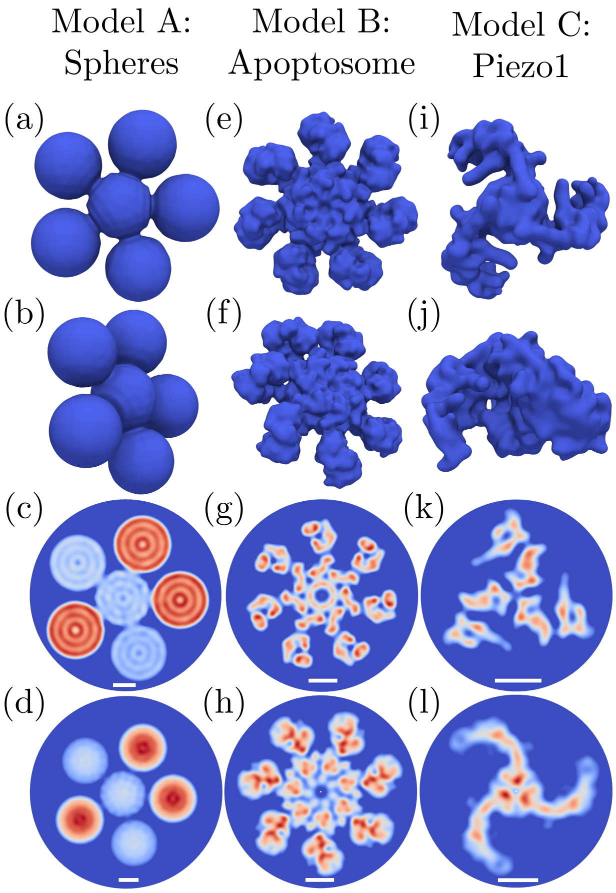

Fig. S5. Averaged reconstructions from the simulated 10-particle scattering patterns, plotted in the same way as in Fig. 7 of the main text.

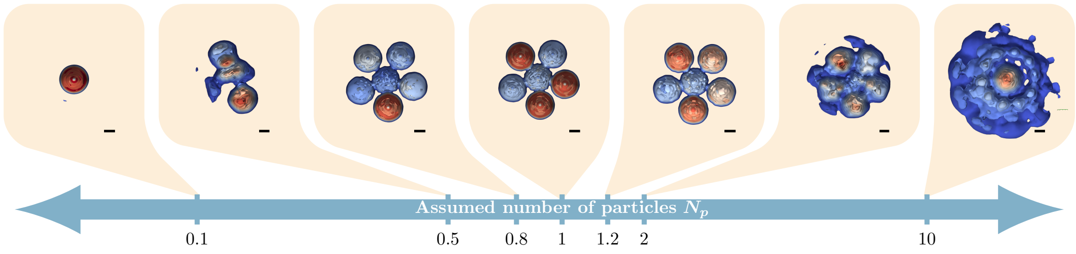

Fig. S6. Reconstructions from *single-particle* scattering data produced while assuming different number of particles N_p in equation (11). Each reconstruction is a result of averaging over 50 independent reconstruction runs. The three averages for $N_\mathrm{p} = 0.8, 1$ and 1.2 are visualized by cutting isosurfaces at 15%, 30%, and 90% of the maximal density, while for $N_\mathrm{p} = 0.1$ and $N_\mathrm{p} = 10$ the isosurface at 15% is plotted. Poorly reconstructed density variations are clearly visible in the averages at $N_\mathrm{p} = 0.8$ and 1.2.

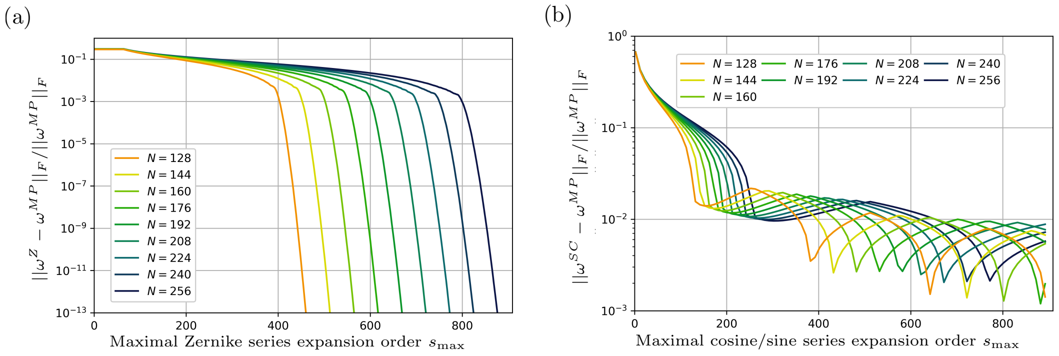

Fig. S7. Relative difference, in Frobenius norm $||\cdot||_{F_q}$, between the weights $\omega^{MP} = \omega_l(p,k)$ generated from the midpoint rule [equation (38c)], and the weights (a) ω^Z from Zernike series approximation [equation (68)], as well as (b) ω^{CS} from cosine/sine series approximation [equations (58)], as function of the considered expansion cutoff s_max. The Frobenius norm of $\omega_l(p,k)$ is given by $\sqrt{\sum_l^L \sum_{p,k}^N |\omega_l(p,k)|^2}$, and was computed for $L = 63$ and various radial grid sizes N specified in the figure legends. The difference between the quadrature weights obtained by different approximations decreases for arbitrary large s_max.

S4. Challenges of experimental measurements and data processing

The FXS approach is suitable for analysing the single-particle ($N_{\rm p} = 1$) and multiple-particle ($N_{\rm p} > 1$) scattering, however these two types of measurements face different experimental challenges. For illustrative purpose, let's consider the 3D case (see Section 2.2 in the main text), while similar arguments apply also in the 2D case. As it follows from equation (11) in the main text, $B_0(q,q')$ for $l = 0$ is scaled by $N_{\rm p}^2$, while all higher-order rotational invariants $B_l(q,q')$ for $l > 0$ are scaled by $N_{\rm p}$. Notice, that $B_0(q,q')$ is directly related to the mean SAXS intensity, and $B_l(q,q')$ for $l > 0$ are related to the angular intensity fluctuations about the mean value. This means that for a large number of particles $N_{\rm p}$ the angular fluctuations become very small compared to the SAXS intensity, which makes their experimental detection very challenging. The ratio $|B_l(q,q')|/B_0(q,q')$ (for $l > 0$) is maximal for $N_{\rm p} = 1$, therefore, the single-particle case represents the "easiest" situation in terms of measuring higher order rotational invariants. However, such single-particle measurements might be very difficult to perform in solution, where solvent scattering becomes a limiting factor. Therefore, moving to the multiple-particle case ($N_{\rm p} > 1$) might be necessary to enhance scattering from particles. Thin cylindrical and sheet liquid jets might be of great help in this case to reduce background scattering from solvent. The general recommendation is still to keep the number of illuminated particles small enough to preserve high angular fluctuations. This requires tight X-ray focusing and high photon flux. It is known that XFEL pulses have fluctuating intensity profiles, which can be further shaped by the applied focusing optics. This means that not all illuminated particles are exposed to the same X-ray intensity, and partially irradiated particles may also exist. These factors will contribute to the uncertainty in the number of illuminated particles $N_{\rm p}$ in each XFEL shapshot (see Section S3), therefore careful analysis will be required to estimate these effects on the extracted rotational invariants. In the case of

single-particle measurements the effects due to finite beam size and incident intensity fluctuations can be mitigated by selecting strong hits and normalizing each diffraction pattern by the incident intensity.

The effect of particle concentration should be estimated in the multiple-particle measurements, since inter-particle interference can distort the measured CCFs. Inter-particle interference manifests itself in the form of relatively sharp peaks in the cross-correlation function $C_M(q, q', \Delta)$ at $q = q'$, $\Delta = 0$ and $\Delta = \pi$, when the average inter-particle distance in a multiple-particle system approaches the particle size. Therefore, the sample should be sufficiently dilute to avoid the undesirable interference effects. Following a usual practice of conventional SAXS measurements seems to be an appropriate strategy. A common approach in SAXS is to perform concentration series measurements, which allow one to detect the interference effects in the low-q region of the SAXS curves. The same approach should be applicable in FXS to determine $B_0(q, q')$ without undesirable interference effects, which is directly related to SAXS profiles. By monitoring the behaviour of the CCF at the specified locations ($q = q'$, $\Delta = 0$ and $\Delta = \pi$) measured at different concentrations, it should be possible to estimate the magnitude of the interference effects and choose a suitable concentration. It is also known, that (both in the case of single- and multiple-particle X-ray scattering) the CCF $C_M(q, q', \Delta)$ has generally higher noise contribution at $q = q'$ and $\Delta = 0$ due to self-correlation of noise. Binning the sufficiently oversampled diffraction patterns helps to reduce the effect of shot noise. At flat Ewald sphere conditions and the absence of inter-particle interference, it is possible to use the symmetry property of the CCF, $C_M(q, q', \Delta) = C_M(q, q', \Delta + \pi)$, to replace the function value in the noisy region.

Another practical problem is particle heterogeneity/polydispersity, which is naturally present in many types of samples and may impact the resolution of the FXS

reconstructions, or even prevent successful reconstructions. While heterogeneity/polydispersity can be tolerated up to some degree (see e.g., (Kurta *et al.*, 2017)), it unavoidably effects the CCFs in the case of multiple-particle scattering measurements. Single-particle measurements are advantageous in this respect, providing a possibility to classify diffraction patterns corresponding to different particle sizes/conformations, and perform correlation analysis and structure reconstructions for each particle class individually.

Various types of systematic errors (e.g., missing data, various detector artefacts, uncompensated background scattering) can affect the CCF in a specific way, both in the case of single-particle and multiple-particle X-ray measurements. FXS can tolerate quite a lot of missing data on individual diffraction patterns by compensating for the lack of data by measuring more diffraction patterns. More specifically, for the central missing region similar rules apply as in conventional CDI. The MTIP algorithm can tolerate partial missing data in the central speckle, but normally fails to achieve successful reconstructions if the entire central speckle is missing. Such situations should be experimentally avoided by carefully considering the detector geometry before the experiment. Other types of missing data (gaps between detectors, masked pixels and extended masked regions) can be tolerated much easier. From the analysis of equation (16) in the main text it follows, that as soon as the denominator (sum of products of the binary mask terms) is nonzero for all values of Δ_t, at given q_k and q_p, the CCF is defined at all points. In other words, this means that it should be possible to successfully determine the CCF if only two unmasked pixels on each diffraction pattern are available (for particular q_k and q_p), while averaging the CCFs over a very large number of measured diffraction patterns. Notice, that such measurements were done in the past by using just two point detectors, see, for instance, (Clark *et al.*, 1983). If the denominator in equation (16) is 0 at given q_k, q_p and Δ_t, the CCF is undefined in this point.

Notice, that depending on the number of contributions in the sum in the denominator of equation (16), the convergence of the CCF $C_M(q_k, q_p, \Delta_t)$ at different points will vary, depending on a particular distribution of the missing data. In practice, the convergence of the CCF can be estimated from the phase maps of the Fourier coefficients of the CCF [see Supplementary in (Kurta *et al.*, 2017)]. The presence of unaccounted systematic detector artefacts/errors may significantly distort the CCF or even prevent its correct determination, especially in the multiple-particle measurements with weak angular fluctuations. Since systematic artefacts are rather case-specific, there is no general recipe and each particular situation may require specific detector corrections. Using more complex forms of the CCF may help to mitigate certain systematic errors and uncompensated background [see, e.g., (Kurta *et al.*, 2017)]. Generally, it is always helpful to perform simulations to estimate the effect of various experimental parameters (e.g., interparticle interference, noise, background, missing detector data, etc). We refer the reader to the following selected publications (Altarelli *et al.*, 2010; Kirian *et al.*, 2011; Kurta *et al.*, 2012; Kurta *et al.*, 2013; Pedrini *et al.*, 2013; Mendez *et al.*, 2016; Martin, 2017; Kurta *et al.*, 2017; Pande *et al.*, 2018), where the effect of various experimental parameters was considered in simulations or experimental data processing.

References

Altarelli, M., Kurta, R. P. & Vartanyants, I. A. (2010). *Phys. Rev. B*, **82**, 104207.

Clark, N. A., Ackerson, B. J. & Hurd, A. J. (1983). *Phys. Rev. Lett.* **50**, 1459.

Kirian, R. A., Schmidt, K. E., Wang, X., Doak, R. B. & Spence, J. C. H. (2011). *Phys. Rev. E*, **84**, 011921.

Kurta, R. P., Altarelli, M., Weckert, E. & Vartanyants, I. A. (2012). *Phys. Rev. B*, **85**, 184204.

Kurta, R. P., Donatelli, J. J., Yoon, C. H., Berntsen, P., Bielecki, J., Daurer, B. J., DeMirci, H., Fromme, P., Hantke, M. F., Maia, F. R. N. C., Munke, A., Nettelblad, C., Pande, K., Reddy, H. K. N., Sellberg, J. A., Sierra, R. G., Svenda, M., van der Schot, G., Vartanyants, I. A., Williams, G. J., Xavier, P. L., Aquila, A., Zwart, P. H. & Mancuso, A. P. (2017). *Phys. Rev. Lett.* **119**, 158102.

Kurta, R. P., Dronyak, R., Altarelli, M., Weckert, E. & Vartanyants, I. A. (2013). *New J. Phys.* **15**, 013059.

Martin, A. V. (2017). *IUCrJ*, **4**, 24–36.

Mendez, D., Watkins, H., Qiao, S., Raines, K. S., Lane, T. J., Schenk, G., Nelson, G., Subramanian, G., Tono, K., Joti, Y., Yabashi, M., Ratnerc, D. & Doniach, S. (2016). *IUCrJ*, **3**, 420–429.

Pande, K., Donatelli, J. J., Malmerberg, E., Foucar, L., Bostedt, C., Schlichting, I. & Zwart, P. H. (2018). *PNAS*, **115**(46), 11772–11777.

Pedrini, B., Menzel, A., Guizar-Sicairos, M., Guzenko, V. A., Gorelick, S., David, C., Patterson, B. D. & Abela, R. (2013). *Nat. Comm.* **4**, 1647.
